# Strategies to implement multifactorial falls prevention interventions in community-dwelling older persons: a systematic review

**DOI:** 10.1186/s13012-022-01257-w

**Published:** 2023-02-06

**Authors:** Sara Vandervelde, Ellen Vlaeyen, Bernadette Dierckx de Casterlé, Johan Flamaing, Sien Valy, Julie Meurrens, Joris Poels, Margot Himpe, Goedele Belaen, Koen Milisen

**Affiliations:** 1 Department of Public Health and Primary Care, Academic Centre for Nursing and Midwifery, Centre of Expertise for Falls and Fracture Prevention Flanders, KU Leuven, Kapucijnenvoer 35 blok d bus 7001, 3000 Leuven, Belgium; 2 Faculty of Medicine and Life Sciences, Hasselt University, Agoralaan, 3590 Diepenbeek, Belgium; 3 Department of Public Health and Primary Care, Academic Centre for Nursing and Midwifery, KU Leuven, Kapucijnenvoer 35 blok d bus 7001, 3000 Leuven, Belgium; 4 Department of Public Health and Primary Care, Gerontology and Geriatrics, Kapucijnenvoer 35 blok d bus 7001, 3000 Leuven, Belgium; 5 Department of Geriatric Medicine, University Hospital Leuven, Herestraat 49, 3000 Leuven, Belgium

**Keywords:** Community setting, Primary health care, Implementation, Practice guidelines, Falls prevention, Aged

## Abstract

**Background:**

One-third of the community-dwelling older persons fall annually. Guidelines recommend the use of multifactorial falls prevention interventions. However, these interventions are difficult to implement into the community. This systematic review aimed to explore strategies used to implement multifactorial falls prevention interventions into the community.

**Methods:**

A systematic search in PubMed (including MEDLINE), CINAHL (EBSCO), Embase, Web of Science (core collection), and Cochrane Library was performed and updated on the 25th of August, 2022. Studies reporting on the evaluation of implementation strategies for multifactorial falls prevention interventions in the community setting were included. Two reviewers independently performed the search, screening, data extraction, and synthesis process (PRISMA flow diagram). The quality of the included reports was appraised by means of a sensitivity analysis, assessing the relevance to the research question and the methodological quality (Mixed Method Appraisal Tool). Implementation strategies were reported according to Proctor et al.’s (2013) guideline for specifying and reporting implementation strategies and the Taxonomy of Behavioral Change Methods of Kok et al. (2016).

**Results:**

Twenty-three reports (eighteen studies) met the inclusion criteria, of which fourteen reports scored high and nine moderate on the sensitivity analysis. All studies combined implementation strategies, addressing different determinants. The most frequently used implementation strategies at individual level were “tailoring,” “active learning,” “personalize risk,” “individualization,” “consciousness raising,” and “participation.” At environmental level, the most often described strategies were “technical assistance,” “use of lay health workers, peer education,” “increasing stakeholder influence,” and “forming coalitions.” The included studies did not describe the implementation strategies in detail, and a variety of labels for implementation strategies were used. Twelve studies used implementation theories, models, and frameworks; no studies described neither the use of a determinant framework nor how the implementation strategy targeted influencing factors.

**Conclusions:**

This review highlights gaps in the detailed description of implementation strategies and the effective use of implementation frameworks, models, and theories. The review found that studies mainly focused on implementation strategies at the level of the older person and healthcare professional, emphasizing the importance of “tailoring,” “consciousness raising,” and “participation” in the implementation process. Studies describing implementation strategies at the level of the organization, community, and policy/society show that “technical assistance,” “actively involving stakeholders,” and “forming coalitions” are important strategies.

**Trial registration:**

PROSPERO CRD42020187450

**Supplementary Information:**

The online version contains supplementary material available at 10.1186/s13012-022-01257-w.

Contribution to the literature

- There is still a knowledge gap in how to implement multifactorial falls prevention interventions into clinical practice.
- The implementation strategies most frequently used at individual level were “tailoring,” “active learning,” “personalize risk,” “individualization,” “consciousness raising,” and “participation.”
- The implementation strategies most often mentioned at environmental level were “technical assistance,” “use of lay health workers, peer education,” “increasing stakeholder influence,” and “forming coalitions.”
- The included studies mainly focused on implementation strategies at the level of the older person and healthcare professional.
- This review recommends using taxonomies and reporting guidelines to select and describe implementation strategies.

## Background

Falls are a major problem in community-dwelling older persons due to their prevalence and consequences. One-third of the older persons living at home (65+) fall annually [1]. Each year, there are 684,000 fatal falls and 37.3 million falls that require medical treatment globally [2]. With an aging population, these numbers will continue to rise [2, 3].

A fall is defined as “an unexpected event in which the older person comes to rest on the ground, floor or lower level” [4]. Each fall is associated with an increased risk of morbidity and mortality and can often lead to physical (e.g., bruises, lacerations, fractures) and psychosocial (e.g., social isolation, fear of falling, depression) consequences [5]. In the USA, falls are the leading cause of injury-related death among persons aged 65 and over [6]. Falls and its related injuries have also a substantial impact on the healthcare cost and the economic burden of society [7]. Consequently, the implementation of effective falls prevention interventions not only may benefit the older person, but it can also reduce the economic burden of society, as shown in literature [8].

Falling is complex, and many factors contribute to its risk (e.g., mobility impairment, medication use, and home hazards) [9]. Due to this complexity, many guidelines recommend to use multifactorial falls prevention interventions [9, 10]. These interventions consist of two or more components tailored to the individual fall-risk profile of the older person [11]. A person older than 65 years is at risk of falling if he or she presents with a fall, reports at least one injurious fall or two or more noninjuries falls, or reports or displays unsteady gait or balance [9, 12, 13]. An older person with high risk of falling receives an assessment of risk factors, i.e., an evaluation of risk factors. Based on the individual fall-risk profile, the person receives an intervention (e.g., one person can receive exercise in combination with recommendations for home hazards; another person can obtain medication advice and supervised exercise) [11]. A Cochrane review supports the “efficacy” of those multifactorial falls prevention interventions in the community setting; it can reduce the rate of falls with 23% compared to usual care or attention control (*RaR* 0.77, 95% *CI* 0.67 to 0.87) [11]. Despite the evidence for the efficacy of these interventions, recent pragmatic cluster-randomized controlled trials, exploring the “effectiveness” of multifactorial falls prevention interventions in the community, found that there is no effect on rate of falls, fall-related injuries, and fractures [14, 15]. It is likely that these differences in results between efficacy and effectiveness testing are due to a poor translation and implementation of multifactorial falls prevention interventions in the community [16].

It is key to address important implementation issues such as the barriers and facilitators (determinants) and select suitable strategies at different levels of the context (i.e., older person, healthcare professional, organization, community, policy/society) to implement multifactorial falls prevention interventions in the community setting (i.e., “home or places of residence that do not provide residential health-related care”) [11, 17, 18]. Currently, research on the implementation of multifactorial falls prevention interventions rarely assesses determinants and derives appropriate implementation strategies (i.e., “a method or technique designed to enhance adoption of a ‘clinical’ intervention”) [19]. In addition, clear and transparent reporting of implementation strategies is scarce [16, 20].

This systematic review aimed to provide an overview of the strategies used to implement multifactorial falls prevention interventions in the community.

## Methods

The review protocol was designed and reported following the PRISMA 2020 statement [21]. This protocol was registered in PROSPERO (CRD42020187450) [22]. The methodology and the main findings of this review were discussed with a multidisciplinary group of 21 stakeholders (e.g., physiotherapists, geriatrician, pharmacist, occupational therapist, registered nurses, policy makers, representatives of older persons, researchers). Purposive sampling was used to compose the stakeholder group (e.g., multidisciplinary group, knowledge about falls prevention, experience with implementation projects in the community). The group met two times to discuss the research question, the included articles, and the results.

### Searches

The search strategy was developed in collaboration with the Biomedical Library, 2Bergen of the University of Leuven, Belgium. The search strategy consisted of three concepts: “older person,” “falls prevention,” and “community setting” (Additional file 1). The search was performed in five databases: PubMed (including MEDLINE), CINAHL (EBSCO), Embase, Web of Science (core collection), and Cochrane Library. The original search was performed from inception until the 18th of May, 2020. On the 25th of August 2022, the researchers updated the search. One researcher (SAV) removed all duplicates in EndNote™, following the de-duplication method of Bramer et al. (2016) [23]. Based on the inclusion and exclusion criteria, two independent reviewers (SAV and JP/GB) screened the titles and abstracts of the records. The reviewers discussed potentially relevant records. After discussion, two reviewers (SAV and SIV/GB) independently read and assessed the reports for eligibility. The reviewers once again discussed the selection process. In addition, the reference lists of the reports, systematic reviews, and meta-analysis were independently reviewed by two researchers (SAV and MH/GB). Discrepancies were resolved by consulting the research group (KM, BDdC, EV, and JF). The selection process was performed in the webtool Rayyan™ and mapped following the PRISMA 2020 flow diagram [21, 24].

### Study inclusion and exclusion criteria

An overview of the inclusion and exclusion criteria can be found in Table 1. Studies reporting on the evaluation of implementation strategies for multifactorial falls prevention interventions in the community setting were included [19]. Multiple publications pertaining the same study were taken into account.

**Table 1:** Inclusion and exclusion criteria

Inclusion criteria	Exclusion criteria
• Evaluation of implementation strategies^a^ for multifactorial falls prevention interventions^b^ in community-dwelling older persons • English, Dutch, and German • Multiple settings (e.g., hospitals, nursing homes) only included if specific information on the community^c^ was available • Experiences, perceptions, and needs of target group (primary research) • Recruitment was done in hospitals, intervention needed to be coordinated in the community^c^	• Implementation strategies^a^ ° Not described ° Not evaluated in the community setting^c^ • Editorials, opinion papers, studies only reported as conference abstract, systematic reviews, meta-analysis • Other settings (e.g., hospitals, nursing homes) • Topics: education (also peer education) was the only implementation strategy, specific population (e.g., frailty, multiple sclerosis, cardiovascular diseases)

^a^Implementation strategy — “A method or technique designed to enhance adoption of a ‘clinical’ intervention” [19]. ^b^Multifactorial falls prevention intervention — “These interventions consist of two or more components tailored to the individual fall risk profile of the older person” [11]. ^c^Community — “Home or places of residence that do not provide residential health-related care” [11]

### Study quality assessment

Two independent reviewers appraised the included reports on their quality by means of a sensitivity analysis (SAV and MH/JM/GB). This analysis took into account the relevance to the research question and the methodological quality of the reports. Table 2 gives an insight in how the sensitivity analysis was assessed. This sensitivity analysis was used to detect reports with a high contribution to the review and high methodological quality, which served as a starting point in the data synthesis (Table 2) [25, 26].

**Table 2:** Results sensitivity analysis

Study	Report	Relevance	Methodological quality	Sensitivity analysis
Study 1	Clemson et al. (2004) [27]	High	High	High
	Ballinger et al. (2006) [28]	High	High	High
Study 2	Mackenzie et al. (2021) [29]	High	High	High
Study 3	Middlebrook et al. (2012) [30]	High	Moderate	High
Study 4	Mora Pinzon et al. (2019) [31]	High	Moderate	High
Study 5	Renehan et al. (2019) [32]	High	Moderate	High
Study 6	Garner et al. (1996) [33]	High	Moderate	High
	Hahn et al. (1996) [34]	High	Moderate	High
	Kempton et al. (2000) [35]	High	Moderate	High
	Barnett et al. (2003) [36]	High	Moderate	High
	Barnett et al. (2004) [37]	High	Low	Moderate
Study 7	Milisen et al. (2006) [38]	High	Moderate	High
Study 8	Mackenzie et al. (2020) [39]	High	Moderate	High
Study 9	Fortinsky et al. (2008) [17]	High	Moderate	High
Study 10	Gholamzadeh et al. (2021) [40]	High	Moderate	High
Study 11	Mahoney et al. (2016) [41]	Moderate	Moderate	Moderate
Study 12	Elley et al. (2008) [42]	Moderate	Moderate	Moderate
Study 13	Kramer et al. (2011) [43]	Moderate	Moderate	Moderate
Study 14	Zimmerman et al. (2017) [44]	Moderate	Moderate	Moderate
Study 15	Schlotthauer et al. (2017) [45]	Moderate	Moderate	Moderate
Study 16	Baker et al. (2007) [46]	High	Low	Moderate
Study 17	Kittipimpanon et al. (2012) [47]	High	Low	Moderate
Study 18	Tiedemann et al. (2021) [48]	High	Low	Moderate

High + high, high. High + moderate, high. Moderate + moderate, moderate. High + low, moderate. Low + low, low

The research and stakeholder group defined, based on the research question and the experiences of the first screening of the reports, five questions to assess the relevance of the reports:

1. Is the implementation strategy clearly described?
2. Is the implementation strategy used in the community?
3. Is the evaluation of an implementation strategy for multifactorial falls prevention interventions in the community described?
4. Does the report measure the effectiveness of the implementation strategy?
5. Does the report explore the experiences with the strategy for the implementation of multifactorial falls prevention interventions?

Based on these items, the relevance of the included reports was scored low, moderate, or high (Additional file 2).

The Mixed Method Appraisal Tool (MMAT) was used to assess the methodological quality of the included reports [49]. The MMAT is designed to appraise methodological quality in systematic mixed studies reviews. The methodological quality of five designs can be appraised: qualitative research, randomized controlled trials, non-randomized studies, quantitative descriptive studies, and mixed methods studies [49]. The tool starts with two screening questions: [1] Are there clear research questions? and [2] Do the collected data allow to address the research questions? The MMAT indicates that further appraisal is not feasible when the answer is “no” or “cannot tell” on one or both screening questions. After the screening questions, the methodological quality of the included reports was assessed based on the study design. For each study design, five specific criteria needed to be rated. The detailed criteria for each design can be found in additional file 2 [49]. The quality of the included reports was scored low, moderate, or high.

### Data extraction strategy

Two reviewers (SAV and MH/JM/GB) independently extracted study characteristics as follows: year, citation, country, source of funding, aim, design, setting, recruitment strategy, sample size, methods of investigation, and analysis. The reviewers also collected data on the characteristics of the target population: age, gender, type of healthcare professional, type of patient, family members, and informal caregiver. In addition, information on the implementation strategies, the multifactorial falls prevention interventions, and follow-up were collected. The Template for Intervention Description and Replication checklist (TIDIeR) was used to describe the multifactorial falls prevention interventions and implementation strategies [50]. All data were compiled in Microsoft Excel™.

### Data synthesis and presentation

Data were summarized in evidence tables, and a narrative synthesis was performed following the “Guidance on the conduct of Narrative synthesis in Systematic Reviews” [51]. To improve conceptual clarity and comprehensiveness, two independent researchers (SAV and GB) synthesized for each report the implementation strategies for the different levels of the context (i.e., older person, healthcare professional, organization, community, policy/society) following the Proctor et al.’s (2013) recommendations for specifying and reporting implementation strategies and Kok et al.’s (2016) Taxonomy of Behaviour Change Methods: an Intervention Mapping approach [18, 52, 53]. The taxonomy of behaviour change methods makes a distinction between behaviour change methods at individual and environmental level [53]. The individual level corresponds to the older person and healthcare professionals. The organization, community, and policy/society are part of the environmental level of the taxonomy. The classification used in this review conforms to the Intervention Mapping approach [18]. The taxonomy of behaviour change methods is part of Intervention Mapping, and it is developed by the same authors [18, 53]. The research group chose to use this taxonomy due to its clear links to theory and determinants of practice for its interventions; it states that a behaviour change method is effective if it meets three conditions: [1] the method needs to target a determinant that predicts behaviour, [2] the method must be able to change the determinant, and [3] the method needs to be translated into a practical application [53]. In addition, the taxonomy of Per Nilsen was used to categorize the implementation theories, models, and frameworks used in the included reports [54]. The reviewers discussed the synthesis, and discrepancies were resolved by consulting the research group (KM, BDdC, EV, and JF).

## Results

**Fig. 1:**
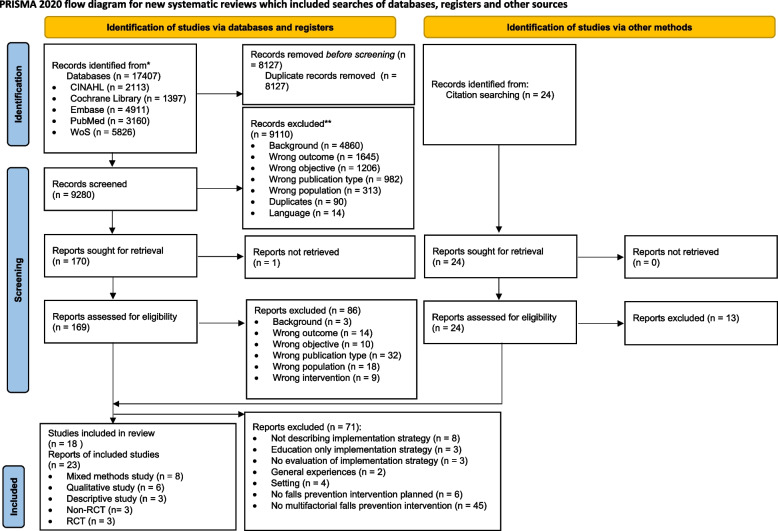
PRISMA flow diagram

The search strategy resulted in a total of 17,407 records, totaling 9280 unique records, after the duplicates were removed. The screening of title and abstract excluded another 9110 records. The full texts of 170 reports were read, of which 83 were found eligible. Eleven additional reports were identified by hand searching fifteen relevant literature reviews and by citation tracking of the eligible reports. In total, 94 reports described the implementation of single, multicomponent, or multifactorial falls prevention interventions. Due to the complexity and the different risk factors that contribute to the risk of falling, the research group and stakeholder group decided to make an amendment to the protocol and to only include reports implementing multifactorial falls prevention interventions. This resulted in the exclusion of 45 reports. After screening the included reports, the researchers and stakeholders noted that some reports (*n* = 11) did not describe or evaluate the implementation strategies. Therefore, it was decided to add the following new inclusion criteria to the protocol: the implementation strategies needed to be described, reports exploring the experiences, and perceptions and needs of the target group were only included if it was primary research. In addition, literature showed that education alone is not sufficient for behaviour change [55]. As a result, an additional exclusion criteria was formulated. Due to the specificity and the complexity of certain diseases like multiple sclerosis, cancer, and cardiovascular diseases, the research group decided not to focus on a specific patient population. Based on all these adaptations, 71 reports were excluded. In total, 23 reports (18 studies) were included in this systematic review [17, 27–48]. A full description of the identification, screening, eligibility, and inclusion process is outlined in the PRISMA 2020 flow diagram (Fig. 1).

### Study quality assessment

The majority of the reports scored high (*n* = 18), and five scored moderate on the relevance to the research question. The methodological quality of the reports was in general moderate (*n* = 16); four reports scored low and three high. No reports were excluded based on the methodological quality. Based on these ratings, the relative contribution (sensitivity analysis) of the reports could be appraised (Table 2). In total, fourteen reports scored high and nine moderate on the sensitivity analysis. Due to the heterogeneity in terms of study design, setting, multifactorial falls prevention interventions, and implementation strategies and outcomes, the extent to which data could be synthesized was limited. Therefore, the results of the sensitivity analysis could not be taken into account in the data synthesis (i.e., giving more weight to reports with a higher score on relevance to the research question and methodological quality).

### Description of studies

Table 3 gives a description of the included studies and reports. Seven studies (twelve reports) were conducted in Australia [27–30, 32–37, 39, 48] and seven studies (seven reports) in the USA [17, 31, 41, 43–46]. The other studies were performed in Belgium [38], New Zealand [42], Iran [40], and Thailand [47]. The majority of the reports (*n* = 15) were older than 5 years [17, 27, 28, 30, 33–38, 41–43, 46, 47]. Seven studies took place in different settings; in a combination of community organization, home of the older persons, senior apartment buildings, and senior centers [27, 28, 31, 40, 41, 45, 47, 48], five studies were performed at the home of the older person [30, 32, 38, 39, 42], two studies took place in a community or senior center [43, 46], one in medical practices [29], and three studies were performed in the community in general [33–37], in home health agencies [17], and in an assisted living community [44]. In total, eight reports used a mixed method design [31, 32, 37–39, 44, 45, 47], six had a qualitative design [28–30, 41, 43, 46], three were quantitative descriptive [17, 33, 36], three were non-randomized controlled trials [34, 35, 48], and three reports were randomized controlled trials [27, 40, 42].

**Table 3:** Description of included studies

Study	Report	Setting	Design	Multifactorial falls prevention Interventions	Implementation strategy	Actor	Action target	Outcomes	Justification
Study 1	Clemson, 2004 [27] Ballinger, 2006 [28]	Community group Home of older person Australia	Clemson • RCT Ballinger • QUAL	Screening: No Assessment: Yes • Group exercise: strength and balance • Medication • Environment: community safety, home hazards • Risk behavior • Vision • Footwear and clothing hazards • Vitamin D and calcium • Hip protectors Control group: • Two social visits from OT students • Not discussing falls or falls prevention	Stepping On Multifaceted community-based program using a small-group learning environment to improve fall self-efficacy, encourage behavioral change, and reduce falls Older person: • 2h weekly session for 7 weeks + 1 follow-up home visit by an OT (6 weeks after final session) + booster session (after 3 months) Healthcare professional: • Training	Older person: • OT Healthcare professional: • Researcher	Older person (70+) Healthcare professionals	Clemson • Effectiveness • Adherence/compliance Ballinger • Satisfaction • Experiences/perception • Attitude	Adult education principles [56] Enhancement of self-efficacy (Bandura) [57] Decision-making process (Janis and Mann) [58]
Study 2	Mackenzie, 2021 [29]	Medical practices Australia	QUAL	Screening: Yes Assessment: Yes • Exercise • Medication review • Orthostatic hypotension • Environment: home hazards • Incontinence • Vision • Podiatry/footwear • Cognitive decline • Falls prevention in general	Integrated solutions for sustainable falls prevention (iSOLVE) • Decision tool for GPs • Stay independent fall checklist for the older person • Fall-risk assessment • List of recommended individualized and tailored interventions • Training of GPs	Older person: • GP Healthcare professional: • Project coordinator	Older person (65+) GPs	Normalization process theory • Feasibility • Penetration • Adoption • Satisfaction • Experiences/perspective • Beliefs	Knowledge-to-action framework (KAT) [59] Behaviour change wheel [60] Normalization process theory [61]
Study 3	Middlebrook, 2012 [30]	Home of older person Australia	QUAL	Screening: Yes Assessment: Yes • Exercise • Medication • Orthostatic hypotension • Environment • Vitamin D • Vision	Chronic disease management plan To offer preventive and coordinated care for older persons with chronic conditions and complex care needs 5 sessions	Older person: • Australian government trough Medicare • GP, PT, OT Healthcare professional: • Government	Older person Healthcare professional	• Acceptability • Sustainability • Challenges/recommendations	No information
Study 4	Mora-Pinzon, 2019 [31]	Community organizations • Multipurpose facility that organizes community activities year round • Apartment complex USA	Mixed Method	Screening: No Assessment: Yes • Group exercise (strength and balance) • Medication • Environment • Risk behavior • Vision • Shoes • Bone health • How to talk to your doctor	Stepping On = Pisando fuerte Cultural linguistically — tailored multifaceted falls prevention program (Hispanic seniors) Multifaceted community-based program using a small-group learning environment to improve fall self-efficacy, encourage behavioral change, and reduce falls 8 sessions of 2,5h + booster session 3 months	Older person: • Leader (allied health professional, fitness expert, community health worker, health educator, promotor, peer leader Healthcare professionals: Researchers Organization: Researchers	Older persons (65+) Healthcare professional Organization	RE-AIM model • Effectiveness • Fidelity • Cost • Reach • Knowledge • Adherence/compliance • Maintenance	Adult education principles [56] Enhancement of self-efficacy (Bandura) [57] Decision-making process (Janis and Mann) [58]
Study 5	Renehan, 2019 [32]	Home of older person Australia	Mixed method	Screening: No Assessment: Yes • Exercise (based on Otago) • Medication • Environment • Vision • Footwear • Education, recommendations	Posthospital falls prevention intervention Tailored exercise program, medication review, education 20- to 30-min exercise program three to five times per week for 6 months Exercise physiologist visited at 1, 2, 4, and 8 weeks Monthly phone calls	PT, RN, pharmacist	Older person (65+)	• Adoption • Challenges and recommendations • Dose • Experiences/perspective • Adherence/compliance • Effectiveness	No information
Study 6	Garner, 1996 [33] Hahn, 1996 [34] Kempton, 2000 [35] Barnett, 2003 [36] Barnett, 2004 [37]	Community setting Australia	Garner, 1996 Descriptive Hahn, 1996 Non-RCT Kempton, 2000 Non-RCT Barnett, 2003 Descriptive Barnett, 2004 Mixed method	Screening: No Assessment: Yes • Group exercise class: insufficient physical activity, poor balance and gait • Medication • Environment: domestic and public environment • Vision • Shoes • Chronic Illness	Stay On Your Feet (SOYF) • Awareness raising and information dissemination • Community education • Policy development • Home safety • Support for GP and health workers	Research group, older persons, local community health teams, community health education groups, community organizations, councils, access committees	Older person(60+) Healthcare professional Organization Community Policy/society	Garner • Penetration • Reach • Challenges and recommendations Hahn • Reach • Effectiveness • Knowledge • Awareness • Attitude Kempton • Reach • Beliefs • Adherence/compliance • Effectiveness • Knowledge • Attitude • Awareness Barnett, 2003 • Penetration • Sustainability • Reach Barnett, 2004 • Sustainability	Goals of the Ottawa Charter for Health promotion (WHO) provided the framework for strategy development [62]
Study 7	Milisen, 2006 [38]	Home of older person Belgium	Mixed method	Screening: Yes Assessment: Yes • Exercise (gait, mobility, balance) • Medication • Orthostatic hypotension • Environment • Risk behavior • Incontinence • Vitamin D • Vision • Podiatry/shoes • Cognitive decline Recommendations and referrals Persons not at risk: Falls prevention in general (leaflet)	Nurse-led multifactorial falls prevention intervention (tailored recommendations) Follow-up 1 month after first visit	Two research nurses	Older person(70+)	• Feasibility • Adoption • Adherence/compliance • timeliness • Experiences/perspective • Reach	No information
Study 8	Mackenzie, 2020 [39]	Home of older person Australia	Mixed method	Screening: yes Assessment: Yes • Exercise (Otago) • Medication • Environment (also community)	Chronic Disease Management Plan To offer preventive and coordinated care for older people with chronic conditions and complex care needs 6 weeks	Older person: • Australian government trough Medicare • GP, PT, OT Healthcare professional: • Government	Older person(75+) Healthcare professional	• Fidelity • Acceptability • Sustainability • Cost • Effectiveness • Timeliness • Recommendations/challenges • Equity • Experiences/perspective • Adherence/compliance • Beliefs	No information
Study 9	Fortinsky, 2008 [17]	Home Health Agencies USA	Descriptive	Screening: Unclear Assessment: Yes • Exercise (mobility and balance) • Medication • Orthostatic hypotension • Environment Conform Yale Frailty and Injuries: Co-operative Studies of Intervention Techniques (FICSIT) trial	Support for implementation & education of healthcare professionals • Implementation team • Training • Supportive material • Champion All 26 HHAs were trained (90min)	Older person: • Home health agencies Healthcare professionals + organization: • Research team, CCFP	Older person(65+) Healthcare professional Organization	• Penetration • Reach	Trans-theoretical model [63] Innovation dissemination theory [64] Behaviour-change strategies
Study 10	Gholamzadeh, 2021 [40]	Health Centers Iran	RCT	Screening: No Assessment: Yes • Group exercise: strength and balance • Medication • Environment: community safety, home hazards • Risk behavior • Vision • Footwear and clothing hazards • Vitamin D and calcium Control group: Stepping On program educative materials in the form of DVDs at the end of the study. They also received a booklet.	Stepping On Multifaceted community-based program using a small-group learning environment to improve fall self-efficacy, encourage behavioral change, and reduce falls Older person: 7 training sessions of 30-60 minutes (2 days a week)	Older person: • Unknown	Older person(65+)	• Effectiveness	Adult education principles [56] Enhancement of self-efficacy (Bandura) [57] Decision-making process (Janis and Mann) [58]
Study 11	Mahoney, 2016 [41]	Senior apartment buildings Home of older person USA	QUAL	Screening: No Assessment: Yes • Group exercise: strength and balance • Medication • Environment: community safety, home hazards • Risk behavior • Vision • Footwear and clothing hazards • Vitamin D and calcium • Hip protectors Info from Clemson, 2004	Stepping On Multifaceted community-based program using a small-group learning environment to improve fall self-efficacy, encourage behavioral change, and reduce falls Older person • 2 h weekly session for 7 weeks + 1 follow-up home visit by an occupational therapist (6 weeks after final session) + booster session (after 3 months) Healthcare professional • Training • Training and program manual	Older person: • RN Healthcare professional: Researcher	Older person (65+) Health care professional	Root cause analysis (RCA) • Fidelity • Adherence/compliance • Belief • Challenges/recommendations	Adult education principles [56] Enhancement of self-efficacy (Bandura) [57] Decision-making process (Janis and Mann) [58]
Study 12	Elley, 2008 [42]	Home of older person • New Zealand	RCT	Screening: Yes Assessment: Yes • Exercise (Otago) • Medication • Orthostatic hypotension • Environment • Incontinence • Vitamin D and calcium • Vision • Footwear • Cognitive decline • Cardiovascular • Musculoskeletal examination	Nurse-led multifactorial falls prevention intervention Older persons: Tailored recommendations Healthcare professionals: Training Control group: social visit + pamphlet	Older person(75+): RN, PT, OT, GP Healthcare professional: RN,researchers	Older person(75+) Healthcare professional	• Penetration • Reach • Effectiveness • Adherence/compliance	No information
Study 13	Kramer, 2011 [43]	Community and senior centers • USA	QUAL	Screening: No Assessment: Yes • Group exercise • Medication • Environment • Cognitive decline	InSTEP A set of three model pilot projects at varying levels of intensity — two each at “high,” “medium,” and “low” Older person: Each model program enrolled small classes for a 12-week program of (a) progressive physical activity classes to improve strength, balance, and gait, (b) home safety evaluation and recommendations for modification, and (c) medical risk assessment and recommendations to discuss any identified risks with a healthcare professional Healthcare professional: • Support implementation • Training Organization: • Training • Support implementation • Collaborations	Older person: Exercise instructor, OT, volunteers, senior center staff, physician, social worker Healthcare professional: Fall Prevention Center of Excellence Organization: Fall Prevention Center of Excellence	Older person Healthcare professional Organization	• Feasibility • Penetration • Awareness • Knowledge • Acceptability • Sustainability • Adoption • Cost • Dose • Challenges/recommendations	Theoretical model based on the extended parallel process model [65]
Study 14	Zimmerman, 2017 [44]	Assisted living communities USA	Mixed method	Screening: Yes Assessment: Yes • Exercise • Medication • Environment • footwear	Assisted Living Falls Prevention and Monitoring Program • Tailored implementation • Support for implementation Control group:In service education session	Researchers	Healthcare professional Organization	• Fidelity • Penetration • Reach • Challenges/recommendations • Effectiveness	No information
Study 15	Schlotthauer, 2017 [45]	Home of older person Independent Living Retirement Community (ILRC) Parks and Recreation Center Parish Nurse Program USA	Mixed method	Screening: No Assessment: Yes • Group exercise: strength and balance • Medication • Environment: community safety, home hazards • Risk behavior • Vision • Footwear and clothing hazards • Vitamin D and calcium • Hip protectors Info from Clemson, 2004	Stepping On Multifaceted community-based program using a small-group learning environment to improve fall self-efficacy, encourage behavioral change, and reduce falls • Independent Living Retirement Community: leader health background versus nonhealth background • Parks and Recreation Center: home visits versus phone calls (follow-up) • Parish Nurse Program: home visits versus phone calls (follow-up)	Older person: Healthcare professional and non healthcare professional Healthcare professional: Researchers	Older person Healthcare professional	• Feasibility • Fidelity • Penetration • Sustainability • Acceptability • Reach • Challenges/recommendations • Experiences/perspectives	Adult education principles [56] Enhancement of self-efficacy (Bandura) [57] Decision-making process (Janis and Mann) [58]
Study 16	Baker, 2007 [46]	Senior center USA	QUAL	Screening: Yes Assessment: Yes • Exercise • Medication • Orthostatic hypotension • Environment • Vision Based on the Yale Frailty and Injury Cooperative Studies of Intervention Trials (known as the Yale FICSIT)	Step by step Support for implementation & education of healthcare professionals • Implementation team • Training • Supportive material • Champion	Older person: Senior center staff Healthcare professional: Investigators who had conducted Yale FICSIT (CCFP) Organization: Research team: CCFP investigators	Older person Healthcare professional Organization	• Penetration • Sustainability • Reach • Challenges and recommendations	Trans-theoretical model [63] PDSA cycle[66]
Study 17	Kittipimpanon, 2012 [47]	Community Home of older person Thailand	Mixed method (action research)	Screening: No Assessment: Yes • Group exercise • Medication • Orthostatic hypotension • Environment • Risk behavior • Vision check • Education	Community-based falls prevention program Community development approach • Falls prevention campaign • Fall management system (surveillance fall notification center and environmental hazards management) • Multifactorial risk assessment • Exercise • Home visits	Public health center, public health nurse, identified leaders of the falls prevention teams, zone team leader, the crown property bureau provided support, researchers	Older person (60+) Organization Community	• Sustainability • Satisfaction • Adherence/compliance • Effectiveness	Appreciation-Influence-Control Technique [67] PRECEDE–PROCEED model Community participation [68]
Study 18	Tiedemann, 2021 [48]	Local health districts New South Wales Australia	Non-RCT	Screening: No Assessment: Yes • Group exercise: strength and balance • Medication • Environment: community safety, home hazards • Risk behavior • Vision • Footwear and clothing hazards • Vitamin D and calcium • Hip protectors Info from Clemson, 2004	Stepping On Multifaceted community-based program using a small-group learning environment to improve fall self-efficacy, encourage behavioral change, and reduce falls Older person • 2h weekly session for 7 weeks + booster session (after 3 months) Healthcare professional • Training	Older person: • OT Healthcare professional: • Stepping On trainer	Older person (65+) Healthcare professional	• Challenges/recommendations • Effectiveness • Satisfaction • Experiences • Motivation • Adherence/compliance • Beliefs	Adult education principles [56] Enhancement of self-efficacy (Bandura) [57] Decision-making process (Janis and Mann) [58]

*GP* General practitioner, *PT* Physiotherapist, *OT* Occupational therapist, *RN* Registered nurse, *HHA* Home health agencies, *CCFP* Connecticut Collaboration for Fall Prevention, PDSA cycle Plan, do, study, act cycle, *QUAL* Qualitative study, *RCT* Randomized controlled trial, Mixed Method mixed-method study, Descriptive, quantitative descriptive study, *Non-RCT* Nonrandomized controlled trial

### Description of multifactorial falls prevention interventions

All included studies implemented multifactorial falls prevention interventions. There is abundant variation in the content and manner in which the multifactorial falls prevention interventions were delivered (e.g., different healthcare professionals involved, supervised versus unsupervised exercise). Table 3 gives an overview of the fall risk factors on which the study interventions focused on. All included reports described the evaluation of risk factors (assessment), but only seven reports (seven studies) clearly described screening for fall risk [29, 30, 38, 39, 42, 44, 46]. All included reports had exercise, medication review/education, and environmental hazards identification/education as part of their intervention. Important fall risk factors such as incontinence, pain, cognitive decline, and fear of falling were often not considered.

### Description of implementation strategies

The majority of the studies described implementation strategies on multiple levels of the context (i.e., older person, healthcare professional, organization, community, policy/society) (*n* = 15) [17, 27–31, 33–37, 39, 41–48]. Renehan et al. (2019), Milisen et al. (2006), and Gholamzadeh et al. (2021) only focused on implementation strategies at the level of the older person [32, 38, 40]. Six studies (seven reports) reported on “Stepping On,” a multifaceted community-based program using a small group learning environment [27, 28, 31, 40, 41, 45, 48]. One study explored the use of iSOLVE (Integrated SOLutions for sustainable falls preVEntion), which consisted of a decision tool for GPs with referrals to other healthcare professionals, a stay independent fall checklist, GP fall risk assessment, and a list of recommended, individualized, and tailored falls prevention interventions [29]. Two studies described a nurse-led multifactorial falls prevention intervention [38, 42]. One study (five reports) reported on “Stay On your Feet,” a large multi strategic program (e.g., awareness raising, education, policy change) [33–37]. Two studies used “Chronic Disease Management” as part of Medicare (formerly Enhanced Primary Care), allowing a general practitioner (GP) to plan and coordinate care for patients with chronic diseases and patients who need multidisciplinary care from a GP and at least two other healthcare professionals [30, 39]. One study described a posthospital tailored multifactorial falls prevention intervention [32], and another study reported on a community-based approach [47]. Four studies described a program that contained external support for implementation (i.e., financial support, support for implementation, and/or training from the research group) [17, 43, 44, 46]. No studies described taxonomies or guidelines to report their implementation strategies.

**Fig. 2:**
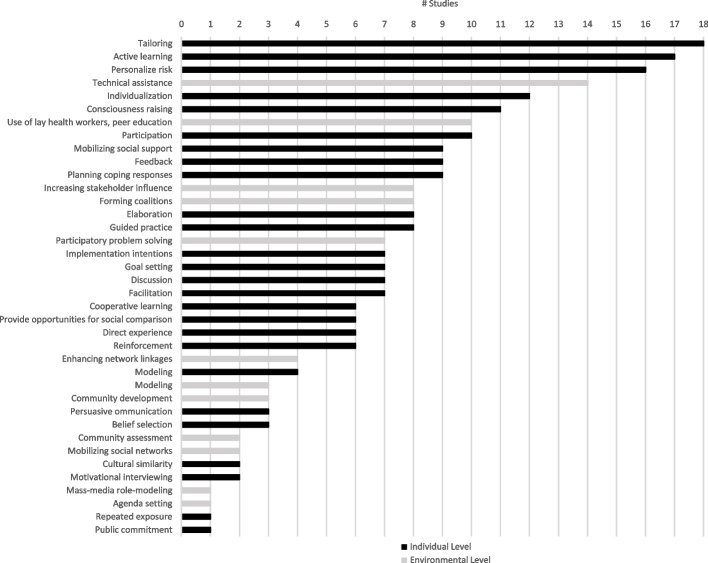
Implementation strategies

Figure 2 gives an overview of the implementation strategies following the “Taxonomy of Behaviour Change Methods; an Intervention Mapping Approach”; a distinction is made between behaviour change methods at individual and environmental level [18, 53]. Some strategies were not only found at individual level but also at the level of the organization, community, and policy/society. An extensive overview of the implementation strategies used in the included reports can be found in additional file 3.

### Individual level

According to the Taxonomy of Behaviour Change Methods, the implementation strategies identified in the included reports aimed to change the following determinants at individual level: “knowledge,” “awareness and risk perception,” ‘habitual, automatic and impulsive behaviors,” “attitudes, beliefs, outcome expectations,” “social influence,” “skills, capabilities and self-efficacy,” and “public stigma” [53].

In total, 26 implementation strategies were identified across eighteen studies (Fig. 2). The most frequently mentioned implementation strategies at individual level were as follows: tailoring (*n* = 18), active learning (*n* = 17), personalize risk (*n* = 16), individualization (*n* = 12), consciousness raising (*n* = 11), and participation (*n* = 10).

The Taxonomy of Behaviour Change Methods defined *tailoring* as *matching the intervention or components to previously measured characteristics of the participant* [53]*.* All reports used tailoring [17, 27–48]. *Personalize risk* entails *providing information about personal costs or risks of action or inaction with respect to target behavior* [53]. Sixteen studies used personalize risk as an implementation strategy [17, 27, 28, 30–43, 45–48]. Tailoring and personalize risk are crucial parts of multifactorial falls prevention interventions, in which two or more components are tailored to the individual fall risk profile of the older person [11]. Tailoring was also used at the level of the healthcare professional (*n* = 9) (e.g., development of tailored tools like referral pads, screening instruments, and tools to plan falls prevention interventions) [27–29, 31, 33–37, 41, 44–46, 48] and by three studies at the level of the organization (e.g., tailored implementation manual) [33–37, 44, 46]. Seventeen studies [17, 27–29, 31–48] described *active learning (i.e., encouraging learning from goal driven and activity based experience)* as an implementation strategy [53]. Active learning was mostly used at the level of the older person and healthcare professionals. Some examples are “Stepping On” [27, 28, 31, 40, 41, 45, 48], “Stay On Your Feet” [33–37], and the “Connecticut Collaboration for Fall Prevention intervention” [17, 46]. “Stepping On” (*n* = 6) used a small group learning environment and incorporated a variety of learning strategies to increase knowledge and competencies (i.e., adult learning principles) [27, 28, 31, 40, 41, 45, 48]. In “Stay On Your Feet,” active learning was part of the community education in which local people were trained as community educators. In addition, older persons were recruited and trained to fulfill three roles: [1] falls prevention advisors [2], home safety advisors, and [3] medication advisors [33–37]. Two studies described the “Connecticut Collaboration for Fall Prevention intervention” that consisted of a multidisciplinary team that trained and encouraged professional behavioral change of healthcare professionals in home health agencies or senior centers [17, 46]. Six studies did not describe the techniques that were used in the educational part of the programs [29, 32, 38, 39, 42, 44]. *Individualization* is defined as *providing opportunities for learners to have personal questions answered or instructions paced according to their individual progress* [53]. Individualization was used in twelve studies at the level of the older person, manifesting in follow-up of recommendations [27, 28, 30–32, 38–42, 45, 46, 48]. In total, eleven studies [27–29, 31, 33–38, 40, 41, 45–48] used *consciousness raising (i.e., providing information, feedback or confrontation about the causes, consequences and alternatives for a problem or a problem behavior)* as an implementation strategy [53]. “Stepping On” [27, 28, 31, 40, 41, 45, 48] and Milisen et al. (2006) [38] raised consciousness among older persons by using healthcare professionals to inform the older persons about their fall risk factors. Although Kok and colleagues categorized consciousness raising only at the individual level, the intervention was also identified at the levels of the organizations, community, policy/society [53]. Examples for these levels are “Stay On Your Feet” and the study of Kittipimpanon et al. (2012) [33–37, 47]. In “Stay On Your Feet,” mass media strategies (i.e., television advertisement, local newspapers, local radio) were used to increase public interest [33–37]. Kittipimpanon and colleagues developed a yearly campaign that consisted of advertisement for their falls prevention program (e.g., polo shirts, stickers) [47]. *Participation* is described as ‘assuring high level engagement of the participants’ group in problem-solving, decision-making, and change activities; with highest level being control by the participants’ group [53]. In total, ten studies described strategies that fit this definition. Participation is mostly used at the level of the older person [27, 28, 31, 33–38, 40, 41, 45–48]. Participation was an important implementation strategy in “Stepping On” [27, 28, 31, 40, 41, 45, 48]. “Stepping On” aims to facilitate older persons to take control, assess coping behaviors, and motivate them to integrate falls prevention interventions in their daily life. In Baker et al. (2007) [46], participation was described at the level of the older person and healthcare professionals. The programs was collectively developed with the older persons and healthcare professionals [46].

### Environmental level

According to the Taxonomy of Behaviour Change Methods, the implementation strategies identified in the included reports aimed to change the following determinants on the environmental level: “social norms,” “social support and social networks,” “change organizations,” “change communities,” and “policy” [53].

In total, twelve implementation strategies were identified across eighteen studies (Fig. 2). The most frequently mentioned implementation strategies at environmental level were as follows: technical assistance (*n* = 14), use of lay health workers, peer education (*n* = 10), increasing stakeholders influence (*n* = 8), forming coalitions (*n* = 8), and participatory problem-solving (*n* = 7).

The Taxonomy of Behaviour Change Methods defined *technical assistance* as *providing technical means to achieve desired behavior* [53]. Fourteen studies used technical assistance as an implementation strategy, including training of the program deliverers (actors) and development and dissemination of supportive materials and tools (e.g., handbooks, flyers, assessment tools) [17, 27–31, 33–37, 39, 41–46, 48]. In addition, three studies offered financial support to healthcare professionals and older persons [33–37, 39, 43]. The strategy *use of lay health workers and peer education* (i.e., *mobilizing members of the target population to serve as boundary spanners, credible sources of information and role models)* [53] had been applied by ten studies [17, 27, 28, 31, 33–37, 41, 44–48]. “Stepping On” and “Stay On Your Feet” involved older persons in the educational component of their program (e.g., peer coleader, providing training) [27, 28, 31, 33–37, 40, 41, 45, 48]. In “Step by Step,” the researchers recruited nurses, experienced in providing community care, and near age peers with the senior center population, as interventionists [46]. Kittipimpanon et al. (2012) involved community members (e.g., housewives, members of a senior club) in the program delivery [47]. In total, eight studies used *increasing stakeholders influence (i.e., increase stakeholder power, legitimacy, and urgency, often by forming coalitions and using community development and social action to change an organization’s policies)* [53] and *forming coalitions (i.e., forming an alliance among individuals or organizations, during which they cooperate in joint action to reach a goal in their own self-interest)* [53] as implementation strategies [29, 30, 33–37, 39, 43, 44, 46, 47]. Middlebrook et al. (2012) and Mackenzie et al. (2020) utilized “chronic disease management” to offer preventive and coordinated care for older persons. General practitioners compiled a multidisciplinary plan, together with occupational therapists and physiotherapists [30, 39]. Developing effective partnerships and networks was also an important component of “Stay On Your Feet” and the study of Kittipimpanon et al. (2012). The researchers cooperated with health organizations, healthcare professionals, intersectoral organizations, and local councils [33–37, 47]. For the development and evaluation of “InSTEP” and “Step by Step,” there was a coalition between centers of expertise in falls prevention, organizations, policy makers, and universities [43, 46]. Seven studies [31, 33–37, 41, 43, 44, 46, 47] used *participatory problem-solving* (i.e., “diagnosing the problem, generating potential solutions, developing priorities, making an action plan and obtaining feedback after implementing the plan”) [53]. The majority of the studies (*n* = 4) that used participatory problem-solving involved stakeholders (e.g., older persons, organizations, policy makers, healthcare professional) to develop, evaluate, and revise their program [33–37, 43, 46, 47]. In three studies, the end users were consulted to translate an existing program to their context [31, 41, 44].

### Additional implementation strategies

The included studies described additional implementation strategies which could not be categorized according the Taxonomy of Behaviour Change Methods [53]. Elley et al. (2008) and Milisen et al. (2006) described a nurse-led multifactorial falls prevention intervention where the *coordination of care* and follow-up was done by one person, a registered nurse [38, 42]. In the two studies using *chronic disease management*, the program was set up by the Australian government allowing older persons, with multiple health problems that require multidisciplinary care, to have five Medicare funded allied health services per year [30, 39].

### Implementation theories, models, and frameworks

Twelve studies used theories, models, or frameworks to develop or evaluate the programs (see Table 4) [17, 27–29, 31, 33–37, 40, 41, 43, 45–48]. First, several classic theories that originate from different fields (e.g., psychology, sociology) were identified. To increase knowledge, “Stepping On” used adult education principles [56], and “InSTEP” used the extended parallel process model [27, 28, 31, 40, 41, 43, 45, 48, 65]. In addition, “Stepping On” [27, 28, 31, 40, 41, 45, 48] used the self-efficacy theory of Bandura [57] and the decision-making process of Janis and Mann [58]. Three studies [17, 29, 46] used the transtheoretical model of Prochaska [63], and Kittipimpanon et al. (2012) used the Appreciation-Influence-Control Technique [67] in a workshop to involve stakeholders [47]. Next, the process models describe the different stages in the translation of research into practice. In this systematic review, three process models were identified: the Ottawa Charter for Health promotion (WHO) [33–37, 62], the plan-do-study-act cycle [46, 66], and the knowledge-to-action framework [29, 59]. In addition, two studies used an implementation theory. In the study of Fortinsky et al. (2008), the innovation dissemination theory of Berwick [64] was used to achieve organizational change [17]. In the iSOLVE project, the Behaviour Change Wheel and normalization process theory were used as implementation theories [29, 60, 61]. The PRECEDE–PROCEED framework [68] was identified in one study and can be categorized as an evaluation framework [47]. In addition, Mora Pinzon et al. [31] used the RE-AIM framework [69], and Mahoney et al. [41] used a root cause analysis to evaluate the implementation project. Lastly, no determinant frameworks were described in the included studies.

**Table 4:** Implementation theories, models, and framework (taxonomy Per Nilsen) [54]

Classic theories	Adult education principles (Field et al.) [56] Extended parallel process model (Witte) [65] Enhancement of self-efficacy (Bandura) [57] Decision-making process (Janis and Mann) [58] Transtheoretical model (Prochaska) [63] Appreciation-Influence-Control Technique (Smith) [67]
Process models	Ottawa Charter for Health promotion (WHO) [62] Plan-do-study-act cycle (Berwick) [66] Knowledge-to-action framework (KAT) [59]
Implementation theories	Innovation dissemination theory (Berwick) [64] Behaviour change wheel [60] Normalization process theory [61]
Evaluation frameworks	PRECEDE–PROCEED (Green) [68] RE-AIM framework [69] Root cause analysis [41]
Determinant frameworks	

## Discussion

Eighteen studies (twenty-three reports) evaluating strategies for the implementation of multifactorial falls prevention interventions in community-dwelling older persons were included in this review. Unlike previous research, this review did not focus on the effectiveness of the falls prevention interventions [11]. It focused on the inconsistency between efficacy and effectiveness testing by gaining insight into how multifactorial falls prevention interventions were currently translated into clinical practice. We explored the strategies used to implement multifactorial falls prevention interventions in the community and provided a synthesis of the implementation strategies following the “Taxonomy of Behaviour Change Methods; an Intervention Mapping Approach” [53].

The majority of the studies described implementation strategies on multiple levels of the context (i.e., older person, healthcare professional, organization, community, policy/society). It is remarkable that implementation strategies were mainly described at the level of the older person and healthcare professional (i.e., individual level). At individual level, we notice that combining tailored implementation strategies, active involvement, and participation are often used to implement multifactorial falls prevention interventions. At the level of the organization, community, and policy/society (i.e., environmental level), it is recognizable that technical assistance and stakeholder involvement are key implementation strategies. Furthermore, this systematic review highlights two key findings.

First, we found that the majority of the studies did not describe the multifactorial falls prevention intervention, implementation strategies, and development of the programs in detail. The included studies used a variety of labels for implementation strategies and lacked operational definitions, increasing the difficulty to gain full insight into the underlying mechanisms of actions for behaviour change [52, 53]. No studies described the use of taxonomies or reporting guidelines. To improve the reporting of the content of behaviour change strategies, it is advised to use guidelines (e.g., Proctor et al.’s recommendations for specifying and reporting implementation strategies, the Standards for Reporting Implementation Studies (StaRI) Statement, or the Workgroup for Intervention Development and Evaluation Research (WIDER)) [52, 70–72]. In addition, for conceptual clarity, it is emphasized to use a taxonomy such as the Taxonomy of Behaviour Change Methods, the Expert Recommendations for Implementing Change (ERIC), the Behavior Change Technique Taxonomy, or the Behaviour Change Wheel, to label implementation strategies [52, 53, 73–75]. In this review, the Taxonomy of Behaviour Change Methods of Kok and colleagues was used, due to its clear links to theory and determinants of practice for its interventions [53]. However, we found that the taxonomy did not give a complete overview of implementation strategies. Additional strategies were identified such as “coordination of care” and “support by the government.” There was also no fit with other taxonomies (e.g., Expert Recommendations for Implementing Change (ERIC), the Behavior Change Technique Taxonomy, the Behaviour Change Wheel, EPOC Taxonomy) [52, 53, 73–76]. In addition, we also found that the distinction between individual and environmental level, made in the Taxonomy of Behaviour Change Methods, was often too strict. As mentioned in the results, some strategies were not only found at individual level but also at the level of the organization, community, policy/society (e.g., belief selection, persuasive communication, active learning, tailoring, consciousness raising, and repeated exposure) [53]. The same remark can be made for the strategies on environmental level. They were also found at the level of the older person and healthcare professional (e.g., technical assistance, use of lay health workers, peer education, increasing stakeholder influence). It can be questioned if the distinction between strategies at individual and environmental level is necessary. Other taxonomies with a clear link to theory and determinants such as the Behavior Change Technique Taxonomy do not make such a distinction.

The second key finding is that solely twelve studies used implementation theories, models, and frameworks, and no studies described neither the use of a determinant framework (e.g., TICD checklist, CFIR) nor how the implementation strategy targeted influencing factors. We also found that the twelve studies that used implementation theories, models, or frameworks for program development did not clearly described how the theories were translated in practical applications in a way that maintained the active mechanisms for effectiveness [77, 78]. Studies show that there is a great value in effectively using implementation frameworks, models, and theories [53, 54, 79]. They can provide a uniform language and inform theoretical thinking and the design, conduct, and evaluation of studies. Implementation theories have directional relationships between determinants; therefore, they can guide what can or cannot work. Suboptimal use of implementation frameworks, models, and theories can impact the success of the implementation efforts, resulting in wasted resources, development of inappropriate implementation strategies, and wrong conclusions [53, 54, 79]. A systematic review on the use of theory in the design of implementation strategies concluded that only 22.5% of the included studies used theories [80]. Mixed results in implementation studies are often attributed to either limited or no theoretical underpinning [54]. Implementation is a dynamic and context-specific process. Each level of the context demands individual tailoring of implementation strategies. Therefore, assessment of influencing factors by means of a determinant framework is crucial, including using the results to select suitable theories and adapt implementation strategies for the specific context [53, 54, 79]. An example of a systematic approach to plan a health promotion program is the study of Vandervelde et al. (2021) on reducing the use of physical restraints in home care. The authors developed and evaluated a multicomponent program to support the implementation of a guideline [81]. By using intervention mapping, they ensured that the program was theoretical, empirical, and practical grounded. During this process, the authors obtained insight into the problem, the behaviour of healthcare professionals, the environment, and the determinants. Together with a stakeholder group, the authors selected theory and evidence-based methods to influence selected determinants; those methods were translated into practical applications (e.g., flyer, tutorials, ambassador for restraint-free home care) [81]. This review found that in falls prevention research, there is still a gap in the detailed description of implementation strategies and the effective use of implementation frameworks, models, and theories, making it difficult to know what does and does not work and to compare and replicate studies.

An important strength is the methodological rigor in which this systematic review was carried out. After all, a comprehensive search strategy was developed in close collaboration with experts of the biomedical library of the university. In addition, during this whole process, the PRISMA statement was followed [21]. A narrative synthesis was performed following the “Guidance on the conduct of Narrative synthesis in Systematic Reviews.” Next, the methods and results of this review were discussed with a group of 21 stakeholders. The stakeholder group recognized the study findings and supported the identified implementation strategies (e.g., tailoring, personalize risk, active learning, consciousness raising). Lastly, this review followed the Proctor et al.’s (2013) recommendations for specifying and reporting implementation strategies and the Taxonomy of Behavioral Change Methods [52, 53]. In addition, TIDIeR was used to extract data on the multifactorial falls prevention interventions and implementation strategies, and the taxonomy of Per Nilsen was used to categorize the implementation theories, models, and frameworks [50, 54]. As already mentioned, the use of guidelines and taxonomies improves conceptual clarity, comprehensiveness, and study replication [20].

This review has some limitations. Despite a comprehensive search strategy, we did identify additional studies from reference lists of systematic reviews and included reports (see Fig. 1). A possible explanation is that we did not search for gray literature, and we did not perform forward snowballing. It is possible that studies were missed. Another limitation is the possibility of publication bias. It is likely that studies with negative results were not published. Lastly, heterogeneity was high in terms of study design, setting, multifactorial falls prevention interventions, implementation strategies, and outcomes. This heterogeneity limited the extent to which data could be synthesized. In addition, the level of description of the implementation strategies used in the reports was poor. This has complicated the categorization of implementation strategies following the Taxonomy of Behaviour Change Methods [53]. To impede this limitation, two researchers categorized the implementations strategies independently. Due to the heterogeneity, we could not take the results of the sensitivity analysis into account in the data synthesis (i.e., giving more weight to reports with a higher score on relevance to the research question and methodological quality).

## Conclusions

This systematic review highlights gaps in the detailed description of implementation strategies and the effective use of implementation frameworks, models, and theories; this can be resolved by using reporting guidelines and taxonomies. In addition, the review found that studies mainly focused on implementation strategies at the level of the older person and healthcare professional. These studies emphasize the importance of tailoring, consciousness raising, and participation in the implementation process of multifactorial falls prevention interventions. Studies using implementation strategies at the level of the organization, community, and policy/society show that technical assistance, actively involving stakeholders and forming coalitions, are important strategies.

## Supplementary Information

**Additional file 1.** Search strategy.

**Additional file 2.** Sensitivity analysis.

**Additional file 3.** Table Implementation strategies.

## Data Availability

All data generated or analyzed during this study are included in this published article and its additional files.

## Abbreviations

GP: General practitioner
MMAT: Mixed Method Appraisal Tool
TIDIeR: Template for Intervention Description and Replication checklist
ESRC: Economic and Social Research Council
iSOLVE: Integrated SOLutions for sustainable falls prevention
CCFP: Connecticut Collaboration for Fall Prevention
StaRI: Standards for Reporting Implementation Studies
WIDER: Workgroup for Intervention Development and Evaluation Research
MeSH: Medical Subject Headings
