# Supplementary material for: Strategies to implement multifactorial falls prevention interventions in community-dwelling older persons: a systematic review
Source: Implement Sci. 2023 Feb 6;18:4. doi: 10.1186/s13012-022-01257-w (PMC9901093; doi:10.1186/s13012-022-01257-w)
Supplement: Supplementary file 1 — Additional file 1. Search strategy. [file 13012_2022_1257_MOESM1_ESM.docx]

# Additional file 1: Search strategy 18/05/2020 (update 25/08/2022)

# PubMed

## Concept 1: Older person

## (((("Aged"[Mesh] OR "Aging"[Mesh:NoExp] OR "Cognitive Aging"[Mesh])) OR (Aged*[tiab] OR Advanced-age*[tiab] OR “Geriatric”[tiab] OR Senior*[tiab] OR 65[tiab] OR 70[tiab] OR 75[tiab] OR 80[tiab] OR 85[tiab] OR 90[tiab] OR 95[tiab] OR 100[tiab] OR Advancing-year*[tiab] OR “Ageing”[tiab] OR “Aging”[tiab] OR Geronto*[tiab] OR Declining-year*[tiab] OR Feeble*[tiab] OR “Of age”[tiab] OR Old-adult*[tiab] OR Older-adult*[tiab] OR “Ripe age”[tiab] OR Senil*[tiab] OR 65-75[tiab] OR 65-80[tiab] OR 65-85[tiab] OR 65-90[tiab] OR 65-95[tiab] OR 65-100[tiab] OR 70-75[tiab] OR 75-80[tiab] OR 75-85[tiab] OR 75-90[tiab] OR 75-95[tiab] OR 75-100[tiab] OR 80-85[tiab] OR 85-90[tiab] OR 85-95[tiab] OR 85-100[tiab] OR 90-95[tiab] OR 90-100[tiab] OR 95-100[tiab])) OR (Frail*[tiab] OR Elder*[tiab] OR Nonagerian*[tiab] OR Octogenarian*[tiab] OR Centarian*[tiab] OR “Senescence”[tiab] OR “Oldest-old”[tiab])) OR (“Longevity”[tiab] OR "Longevity"[Mesh])

## Concept 2: Community setting

((((("Independent Living"[Mesh] OR "Community Health Services"[Mesh:NoExp] OR "Adult Day Care Centers"[Mesh] OR "Community Health Nursing"[Mesh:NoExp] OR "Home Health Nursing"[Mesh] OR "Community Networks"[Mesh] OR "Community Participation"[Mesh] OR "Home Care Services"[Mesh:NoExp] OR "Home Nursing"[Mesh] OR "Homemaker Services"[Mesh] OR "Senior Centers"[Mesh] OR "Community Medicine"[Mesh] OR "Ambulatory Care Facilities"[Mesh:NoExp] OR "Community Health Centers"[Mesh:NoExp] OR "Primary Health Care"[Mesh:NoExp] OR "Home Care Agencies"[Mesh] OR "Health Services for the Aged"[Mesh] OR "Assisted Living Facilities"[Mesh] OR "Housing for the Elderly"[Mesh])) OR ("Independent Living"[tiab] OR Adult-Day-Care-Center*[tiab] OR "Home Health Nursing"[tiab] OR Community-Network*[tiab] OR "Community Participation"[tiab] OR "Home Nursing"[tiab] OR Homemaker-Service*[tiab] OR Senior-Cent*[tiab] OR "Community Medicine"[tiab] OR “Community-care”[tiab] OR Ambulatory-Care-Facilit*[tiab] OR "Primary Health Care"[tiab] OR “Primary care”[tiab] OR Home-health-servic*[tiab] OR "Health Services for the Aged"[tiab] OR "Housing for the Elderly"[tiab])) OR (Community-Dwelling*[tiab] OR “Aging in Place”[tiab] OR Community-Health*[tiab] OR “Home Health Care”[tiab] OR home-healthcare[tiab] OR Community-Involvement*[tiab] OR “Public Participation”[tiab] OR Community-Action*[tiab] OR “Domiciliary Care”[tiab] OR “Home Care”[tiab] OR “Centers for the Aged”[tiab] OR Ambulatory-Health-Cent*[tiab] OR Neighborhood-Health-Cent*[tiab] OR Neighbourhood-Health-Cent*[tiab] OR “Primary Healthcare”[tiab] OR Home-Health-Agenc*[tiab] OR Geriatric-Health-Servic*[tiab] OR Assisted-Living-Facilit*[tiab])) OR (“Home visit”[tiab] OR “visiting nursing service”[tiab] OR outpatient-department*[tiab] OR “elderly care”[tiab] OR “old age assistance”[tiab] OR “primary nursing care”[tiab] OR “first line care”[tiab] OR visiting-nursing-station*[tiab] OR visiting-nurse-station*[tiab] OR visiting-nurse-service*[tiab] OR visiting-nursing-service*[tiab] OR house-call*[tiab] OR home-visit*[tiab] OR home-health-agenc*[tiab] OR “homecare”[tiab] OR home-treatment*[tiab] OR home-service*[tiab] OR “home help”[tiab] OR “domestic health care”[tiab] OR “domestic healthcare”[tiab] OR “public health nursing”[tiab] OR “district nursing”[tiab] OR “community nursing”[tiab] OR “community based nursing”[tiab])) OR (“Community living”[tiab] OR “Assisted Living”[tiab] OR "Community Reintegration”[tiab] OR "Home Occupational Therapy"[tiab] OR "Home Physical Therapy"[tiab] OR "Home Rehabilitation"[tiab] OR "Respite Care"[tiab] OR Preventive-Health*[tiab] OR "Preventive Health Services"[Mesh:NoExp] OR "Gerontologic Care”[tiab])

## Concept 3: Falls prevention

(Fall*[tiab] OR slip*[tiab] OR "Accidental Falls"[Mesh]) AND ("Accident Prevention"[Mesh:NoExp] OR "Safety"[Mesh:NoExp] OR prevent*[tiab] OR safet*[tiab])

## Total search strategy

((((((("Aged"[Mesh] OR "Aging"[Mesh:NoExp] OR "Cognitive Aging"[Mesh])) OR (Aged*[tiab] OR Advanced-age*[tiab] OR “Geriatric”[tiab] OR Senior*[tiab] OR 65[tiab] OR 70[tiab] OR 75[tiab] OR 80[tiab] OR 85[tiab] OR 90[tiab] OR 95[tiab] OR 100[tiab] OR Advancing-year*[tiab] OR “Ageing”[tiab] OR “Aging”[tiab] OR Geronto*[tiab] OR Declining-year*[tiab] OR Feeble*[tiab] OR “Of age”[tiab] OR Old-adult*[tiab] OR Older-adult*[tiab] OR “Ripe age”[tiab] OR Senil*[tiab] OR 65-75[tiab] OR 65-80[tiab] OR 65-85[tiab] OR 65-90[tiab] OR 65-95[tiab] OR 65-100[tiab] OR 70-75[tiab] OR 75-80[tiab] OR 75-85[tiab] OR 75-90[tiab] OR 75-95[tiab] OR 75-100[tiab] OR 80-85[tiab] OR 85-90[tiab] OR 85-95[tiab] OR 85-100[tiab] OR 90-95[tiab] OR 90-100[tiab] OR 95-100[tiab])) OR (Frail*[tiab] OR Elder*[tiab] OR Nonagerian*[tiab] OR Octogenarian*[tiab] OR Centarian*[tiab] OR “Senescence”[tiab] OR “Oldest-old”[tiab])) OR (“Longevity”[tiab] OR "Longevity"[Mesh]))) AND (((((("Independent Living"[Mesh] OR "Community Health Services"[Mesh:NoExp] OR "Adult Day Care Centers"[Mesh] OR "Community Health Nursing"[Mesh:NoExp] OR "Home Health Nursing"[Mesh] OR "Community Networks"[Mesh] OR "Community Participation"[Mesh] OR "Home Care Services"[Mesh:NoExp] OR "Home Nursing"[Mesh] OR "Homemaker Services"[Mesh] OR "Senior Centers"[Mesh] OR "Community Medicine"[Mesh] OR "Ambulatory Care Facilities"[Mesh:NoExp] OR "Community Health Centers"[Mesh:NoExp] OR "Primary Health Care"[Mesh:NoExp] OR "Home Care Agencies"[Mesh] OR "Health Services for the Aged"[Mesh] OR "Assisted Living Facilities"[Mesh] OR "Housing for the Elderly"[Mesh])) OR ("Independent Living"[tiab] OR Adult-Day-Care-Center*[tiab] OR "Home Health Nursing"[tiab] OR Community-Network*[tiab] OR "Community Participation"[tiab] OR "Home Nursing"[tiab] OR Homemaker-Service*[tiab] OR Senior-Cent*[tiab] OR "Community Medicine"[tiab] OR “Community-care”[tiab] OR Ambulatory-Care-Facilit*[tiab] OR "Primary Health Care"[tiab] OR “Primary care”[tiab] OR Home-health-servic*[tiab] OR "Health Services for the Aged"[tiab] OR "Housing for the Elderly"[tiab])) OR (Community-Dwelling*[tiab] OR “Aging in Place”[tiab] OR Community-Health*[tiab] OR “Home Health Care”[tiab] OR home-healthcare[tiab] OR Community-Involvement*[tiab] OR “Public Participation”[tiab] OR Community-Action*[tiab] OR “Domiciliary Care”[tiab] OR “Home Care”[tiab] OR “Centers for the Aged”[tiab] OR Ambulatory-Health-Cent*[tiab] OR Neighborhood-Health-Cent*[tiab] OR Neighbourhood-Health-Cent*[tiab] OR “Primary Healthcare”[tiab] OR Home-Health-Agenc*[tiab] OR Geriatric-Health-Servic*[tiab] OR Assisted-Living-Facilit*[tiab])) OR (“Home visit”[tiab] OR “visiting nursing service”[tiab] OR outpatient-department*[tiab] OR “elderly care”[tiab] OR “old age assistance”[tiab] OR “primary nursing care”[tiab] OR “first line care”[tiab] OR visiting-nursing-station*[tiab] OR visiting-nurse-station*[tiab] OR visiting-nurse-service*[tiab] OR visiting-nursing-service*[tiab] OR house-call*[tiab] OR home-visit*[tiab] OR home-health-agenc*[tiab] OR “homecare”[tiab] OR home-treatment*[tiab] OR home-service*[tiab] OR “home help”[tiab] OR “domestic health care”[tiab] OR “domestic healthcare”[tiab] OR “public health nursing”[tiab] OR “district nursing”[tiab] OR “community nursing”[tiab] OR “community based nursing”[tiab])) OR (“Community living”[tiab] OR “Assisted Living”[tiab] OR "Community Reintegration”[tiab] OR "Home Occupational Therapy"[tiab] OR "Home Physical Therapy"[tiab] OR "Home Rehabilitation"[tiab] OR "Respite Care"[tiab] OR Preventive-Health*[tiab] OR "Preventive Health Services"[Mesh:NoExp] OR "Gerontologic Care”[tiab]))) AND (((Fall*[tiab] OR slip*[tiab] OR "Accidental Falls"[Mesh]) AND ("Accident Prevention"[Mesh:NoExp] OR "Safety"[Mesh:NoExp] OR prevent*[tiab] OR safet*[tiab])))

# Embase

## Concept 1: Older person

## ('aged'/de OR 'aging'/de OR 'senescence'/de OR 'geriatric patient'/exp OR 'frail elderly'/exp OR 'very elderly'/exp) OR ('aged*':ti,ab,kw OR 'elder*':ti,ab,kw OR 'senior*':ti,ab,kw OR 'aging*':ti,ab,kw OR 'senescence':ti,ab,kw OR 'advanced age*':ti,ab,kw OR 'geriatric':ti,ab,kw OR '65':ti,ab,kw OR '70':ti,ab,kw OR '75':ti,ab,kw OR '80':ti,ab,kw OR '85':ti,ab,kw OR '90':ti,ab,kw OR '95':ti,ab,kw OR '100':ti,ab,kw OR 'advancing-year*':ti,ab,kw OR 'ageing':ti,ab,kw OR 'geronto*':ti,ab,kw OR 'declining-year*':ti,ab,kw OR 'feeble*':ti,ab,kw OR 'of age':ti,ab,kw OR 'old adult*':ti,ab,kw OR 'older adult*':ti,ab,kw OR 'ripe age':ti,ab,kw OR 'senil*':ti,ab,kw OR '65?75':ti,ab,kw OR '65?80':ti,ab,kw OR '65?85':ti,ab,kw OR '65?90':ti,ab,kw OR '65?95':ti,ab,kw OR '65?100':ti,ab,kw OR '70?75':ti,ab,kw OR '75?80':ti,ab,kw OR '75?85':ti,ab,kw OR '75?90':ti,ab,kw OR '75?95':ti,ab,kw OR '75?100':ti,ab,kw OR '80?85':ti,ab,kw OR '85?90':ti,ab,kw OR '85?95':ti,ab,kw OR '85?100':ti,ab,kw OR '90?95':ti,ab,kw OR '90?100':ti,ab,kw OR '95?100':ti,ab,kw OR 'frail*':ti,ab,kw OR 'nonagerian*':ti,ab,kw OR 'octogenarian*':ti,ab,kw OR 'centarian*':ti,ab,kw OR 'oldest-old':ti,ab,kw) OR ('longevity'/exp OR 'longevity':ti,ab,kw)

## Concept 2: Community setting

('independent living'/exp OR 'community care'/de OR 'community health nursing'/de OR 'senior center'/exp OR 'home care'/de OR 'home health agency'/exp OR 'home visit'/exp OR 'visiting nursing service'/exp OR 'community participation'/de OR 'community medicine'/exp OR 'outpatient department'/exp OR 'primary health care'/exp OR 'elderly care'/de OR 'assisted living facility'/exp) OR ('independent living':ti,ab,kw OR 'health service* for the elder*':ti,ab,kw OR 'geriatric health service*':ti,ab,kw OR 'neighborhood health cent*':ti,ab,kw OR 'neighbourhood health cent*':ti,ab,kw OR 'ambulatory health cent*':ti,ab,kw OR 'community action*':ti,ab,kw OR 'public participation':ti,ab,kw OR 'community involvement*':ti,ab,kw OR 'community dwelling*':ti,ab,kw OR 'home health servic*':ti,ab,kw OR 'primary care':ti,ab,kw OR 'assisted living':ti,ab,kw OR 'old age assistance':ti,ab,kw OR 'elderly care':ti,ab,kw OR 'health services for the aged':ti,ab,kw OR 'primary nursing care':ti,ab,kw OR 'primary healthcare':ti,ab,kw OR 'first line care':ti,ab,kw OR 'primary health care':ti,ab,kw OR 'community health center*':ti,ab,kw OR 'ambulatory care facilit*':ti,ab,kw OR 'outpatient department*':ti,ab,kw OR 'community medicine':ti,ab,kw OR 'community participation':ti,ab,kw OR 'community network*':ti,ab,kw OR 'visiting nursing station*':ti,ab,kw OR 'visiting nurse station*':ti,ab,kw OR 'visiting nurse service*':ti,ab,kw OR 'visiting nursing service*':ti,ab,kw OR 'house call*':ti,ab,kw OR 'home visit*':ti,ab,kw OR 'home health agenc*':ti,ab,kw OR 'homemaker service*':ti,ab,kw OR 'homecare':ti,ab,kw OR 'home treatment*':ti,ab,kw OR 'home service*':ti,ab,kw OR 'home nursing':ti,ab,kw OR 'home help':ti,ab,kw OR 'home health nursing':ti,ab,kw OR 'home health care':ti,ab,kw OR 'domiciliary care':ti,ab,kw OR 'domestic health care':ti,ab,kw OR 'domestic healthcare':ti,ab,kw OR 'home care':ti,ab,kw OR 'adult day care cent*':ti,ab,kw OR 'senior cent*':ti,ab,kw OR 'cent* for the elderly':ti,ab,kw OR 'cent* for the aged':ti,ab,kw OR 'public health nursing':ti,ab,kw OR 'district nursing':ti,ab,kw OR 'community nursing':ti,ab,kw OR 'community based nursing':ti,ab,kw OR 'community health*':ti,ab,kw OR 'community care':ti,ab,kw OR 'aging in place':ti,ab,kw OR 'housing for the elderly':ti,ab,kw OR ‘home$healthcare’:ti,ab,kw) OR ('community reintegration'/exp OR 'community living'/exp OR 'home rehabilitation'/exp OR 'respite care'/exp OR 'community living':ti,ab,kw OR 'assisted living':ti,ab,kw OR 'community reintegration':ti,ab,kw OR 'home rehabilitation':ti,ab,kw OR 'respite care':ti,ab,kw OR 'preventive health care':ti,ab,kw OR 'gerontologic care':ti,ab,kw)

## Concept 3: Falls prevention

('falling'/exp OR 'fall*':ti,ab,kw OR 'slip':ti,ab,kw) AND ('accident prevention'/exp OR 'safety'/de OR 'prevent*':ti,ab,kw OR 'safet*':ti,ab,kw)

## Total search strategy

(('aged'/de OR 'aging'/de OR 'senescence'/de OR 'geriatric patient'/exp OR 'frail elderly'/exp OR 'very elderly'/exp) OR ('aged*':ti,ab,kw OR 'elder*':ti,ab,kw OR 'senior*':ti,ab,kw OR 'aging*':ti,ab,kw OR 'senescence':ti,ab,kw OR 'advanced age*':ti,ab,kw OR 'geriatric':ti,ab,kw OR '65':ti,ab,kw OR '70':ti,ab,kw OR '75':ti,ab,kw OR '80':ti,ab,kw OR '85':ti,ab,kw OR '90':ti,ab,kw OR '95':ti,ab,kw OR '100':ti,ab,kw OR 'advancing-year*':ti,ab,kw OR 'ageing':ti,ab,kw OR 'geronto*':ti,ab,kw OR 'declining-year*':ti,ab,kw OR 'feeble*':ti,ab,kw OR 'of age':ti,ab,kw OR 'old adult*':ti,ab,kw OR 'older adult*':ti,ab,kw OR 'ripe age':ti,ab,kw OR 'senil*':ti,ab,kw OR '65?75':ti,ab,kw OR '65?80':ti,ab,kw OR '65?85':ti,ab,kw OR '65?90':ti,ab,kw OR '65?95':ti,ab,kw OR '65?100':ti,ab,kw OR '70?75':ti,ab,kw OR '75?80':ti,ab,kw OR '75?85':ti,ab,kw OR '75?90':ti,ab,kw OR '75?95':ti,ab,kw OR '75?100':ti,ab,kw OR '80?85':ti,ab,kw OR '85?90':ti,ab,kw OR '85?95':ti,ab,kw OR '85?100':ti,ab,kw OR '90?95':ti,ab,kw OR '90?100':ti,ab,kw OR '95?100':ti,ab,kw OR 'frail*':ti,ab,kw OR 'nonagerian*':ti,ab,kw OR 'octogenarian*':ti,ab,kw OR 'centarian*':ti,ab,kw OR 'oldest-old':ti,ab,kw) OR ('longevity'/exp OR 'longevity':ti,ab,kw)) AND (('independent living'/exp OR 'community care'/de OR 'community health nursing'/de OR 'senior center'/exp OR 'home care'/de OR 'home health agency'/exp OR 'home visit'/exp OR 'visiting nursing service'/exp OR 'community participation'/de OR 'community medicine'/exp OR 'outpatient department'/exp OR 'primary health care'/exp OR 'elderly care'/de OR 'assisted living facility'/exp) OR ('independent living':ti,ab,kw OR 'health service* for the elder*':ti,ab,kw OR 'geriatric health service*':ti,ab,kw OR 'neighborhood health cent*':ti,ab,kw OR 'neighbourhood health cent*':ti,ab,kw OR 'ambulatory health cent*':ti,ab,kw OR 'community action*':ti,ab,kw OR 'public participation':ti,ab,kw OR 'community involvement*':ti,ab,kw OR 'community dwelling*':ti,ab,kw OR 'home health servic*':ti,ab,kw OR 'primary care':ti,ab,kw OR 'assisted living':ti,ab,kw OR 'old age assistance':ti,ab,kw OR 'elderly care':ti,ab,kw OR 'health services for the aged':ti,ab,kw OR 'primary nursing care':ti,ab,kw OR 'primary healthcare':ti,ab,kw OR 'first line care':ti,ab,kw OR 'primary health care':ti,ab,kw OR 'community health center*':ti,ab,kw OR 'ambulatory care facilit*':ti,ab,kw OR 'outpatient department*':ti,ab,kw OR 'community medicine':ti,ab,kw OR 'community participation':ti,ab,kw OR 'community network*':ti,ab,kw OR 'visiting nursing station*':ti,ab,kw OR 'visiting nurse station*':ti,ab,kw OR 'visiting nurse service*':ti,ab,kw OR 'visiting nursing service*':ti,ab,kw OR 'house call*':ti,ab,kw OR 'home visit*':ti,ab,kw OR 'home health agenc*':ti,ab,kw OR 'homemaker service*':ti,ab,kw OR 'homecare':ti,ab,kw OR 'home treatment*':ti,ab,kw OR 'home service*':ti,ab,kw OR 'home nursing':ti,ab,kw OR 'home help':ti,ab,kw OR 'home health nursing':ti,ab,kw OR 'home health care':ti,ab,kw OR 'domiciliary care':ti,ab,kw OR 'domestic health care':ti,ab,kw OR 'domestic healthcare':ti,ab,kw OR 'home care':ti,ab,kw OR 'adult day care cent*':ti,ab,kw OR 'senior cent*':ti,ab,kw OR 'cent* for the elderly':ti,ab,kw OR 'cent* for the aged':ti,ab,kw OR 'public health nursing':ti,ab,kw OR 'district nursing':ti,ab,kw OR 'community nursing':ti,ab,kw OR 'community based nursing':ti,ab,kw OR 'community health*':ti,ab,kw OR 'community care':ti,ab,kw OR 'aging in place':ti,ab,kw OR 'housing for the elderly':ti,ab,kw OR ‘home$healthcare’:ti,ab,kw) OR ('community reintegration'/exp OR 'community living'/exp OR 'home rehabilitation'/exp OR 'respite care'/exp OR 'community living':ti,ab,kw OR 'assisted living':ti,ab,kw OR 'community reintegration':ti,ab,kw OR 'home rehabilitation':ti,ab,kw OR 'respite care':ti,ab,kw OR 'preventive health care':ti,ab,kw OR 'gerontologic care':ti,ab,kw)) AND (('falling'/exp OR 'fall*':ti,ab,kw OR 'slip':ti,ab,kw) AND ('accident prevention'/exp OR 'safety'/de OR 'prevent*':ti,ab,kw OR 'safet*':ti,ab,kw))

# CINAHL

## Concept 1: Older person

( (MH "Aged") OR (MH "Aged, 80 and Over") OR (MH "Centenarians") OR (MH "Frail Elderly") OR (MH "Aging") OR (MH "Cognitive Aging") OR (MH "Longevity") ) OR ( TI(Aged* OR Advanced-age* OR Geriatric OR Senior* OR 65 OR 70 OR 75 OR 80 OR 85 OR 90 OR 95 OR 100 OR Advancing-year* OR Ageing OR Aging OR Geronto* OR Declining-year* OR Feeble* OR Of age OR Old-adult* OR Older-adult* OR Longevity OR “Ripe age” OR Senil* OR “65-75” OR “65-80” OR “65-85” OR “65-90” OR “65-95” OR “65-100” OR “70-75” OR “75-80” OR “75-85” OR “75-90” OR “75-95” OR “75-100” OR “80-85” OR “85-90” OR “85-95” OR “85-100” OR “90-95” OR “90-100” OR “95-100” OR Frail* OR Elder* OR Nonagerian* OR Octogenarian* OR Centarian* OR Senescence OR “Oldest-old”) OR AB(Aged* OR Advanced-age* OR Geriatric OR Senior* OR 65 OR 70 OR 75 OR 80 OR 85 OR 90 OR 95 OR 100 OR Advancing-year* OR Ageing OR Aging OR Geronto* OR Declining-year* OR Feeble* OR Of age OR Old-adult* OR Older-adult* OR Longevity OR “Ripe age” OR Senil* OR “65-75” OR “65-80” OR “65-85” OR “65-90” OR “65-95” OR “65-100” OR “70-75” OR “75-80” OR “75-85” OR “75-90” OR “75-95” OR “75-100” OR “80-85” OR “85-90” OR “85-95” OR “85-100” OR “90-95” OR “90-100” OR “95-100” OR Frail* OR Elder* OR Nonagerian* OR Octogenarian* OR Centarian* OR Senescence OR “Oldest-old”) )

## Concept 2: Community setting

( (MH "Community Living") OR (MH "Assisted Living") OR (MH "Community Reintegration") OR (MH "Community Health Services") OR (MH "Community Health Nursing") OR (MH "Community Networks") OR (MH "Home Health Care") OR (MH "Home Nursing, Professional") OR (MH "Home Occupational Therapy") OR (MH "Home Physical Therapy") OR (MH "Home Rehabilitation") OR (MH "Respite Care") OR (MH "Preventive Health Care") OR (MH "Senior Centers") OR (MH "Housing for the Elderly") OR (MH "Ambulatory Care Facilities") OR (MH "Community Health Centers") OR (MH "Home Health Agencies") OR (MH "Home Nursing") OR (MH "Gerontologic Care") OR (MH "Homemaker Services") OR (MH "Community Medicine") OR (MH "Primary Health Care") OR (MH "Health Services for the Aged") OR (MH "Housing for the Elderly") ) OR ( TI(“Independent Living” OR Adult-Day-Care-Center* OR “Home Health Nursing” OR Community-Network* OR “Community Participation” OR “Home Nursing” OR Homemaker-Service* OR Senior-Cent* OR “Community Medicine” OR “Community-care” OR Ambulatory-Care-Facilit* OR “Primary Health Care” OR “Primary care” OR Home-health-servic* OR “Health Services for the Aged” OR “Housing for the Elderly” OR Community-Dwelling* OR “Aging in Place” OR Community-Health* OR “Home Health Care” OR “home-healthcare” OR Community-Involvement* OR “Public Participation” OR Community-Action* OR “Domiciliary Care” OR “Home Care” OR “Centers for the Aged” OR Ambulatory-Health-Cent* OR Neighborhood-Health-Cent* OR Neighbourhood-Health-Cent* OR “Primary Healthcare” OR Home-Health-Agenc* OR Geriatric-Health-Servic* OR Assisted-Living-Facilit* OR “Home visit” OR “visiting nursing service” OR outpatient-department* OR “elderly care” OR “old age assistance” OR “primary nursing care” OR “first line care” OR visiting-nursing-station* OR visiting-nurse-station* OR visiting-nurse-service* OR visiting-nursing-service* OR house-call* OR home-visit* OR home-health-agenc* OR “homecare” OR home-treatment* OR home-service* OR “home help” OR “domestic health care” OR “domestic healthcare” OR “public health nursing” OR “district nursing” OR “community nursing” OR “community based nursing” OR “Community Living” OR “Assisted Living” OR “Community Reintegration” OR “Home Occupational Therapy” OR “Home Physical Therapy” OR “Home Rehabilitation” OR “Respite Care” OR “Preventive Health Care” OR “Gerontologic Care”) OR AB(“Independent Living” OR Adult-Day-Care-Center* OR “Home Health Nursing” OR Community-Network* OR “Community Participation” OR “Home Nursing” OR Homemaker-Service* OR Senior-Cent* OR “Community Medicine” OR “Community-care” OR Ambulatory-Care-Facilit* OR “Primary Health Care” OR “Primary care” OR Home-health-servic* OR “Health Services for the Aged” OR “Housing for the Elderly” OR Community-Dwelling* OR “Aging in Place” OR Community-Health* OR “Home Health Care” OR “home-healthcare” OR Community-Involvement* OR “Public Participation” OR Community-Action* OR “Domiciliary Care” OR “Home Care” OR “Centers for the Aged” OR Ambulatory-Health-Cent* OR Neighborhood-Health-Cent* OR “Primary Healthcare” OR Home-Health-Agenc* OR Geriatric-Health-Servic* OR Assisted-Living-Facilit* OR “Home visit” OR “visiting nursing service” OR outpatient-department* OR “elderly care” OR “old age assistance” OR “primary nursing care” OR “first line care” OR visiting-nursing-station* OR visiting-nurse-station* OR visiting-nurse-service* OR visiting-nursing-service* OR house-call* OR home-visit* OR home-health-agenc* OR “homecare” OR home-treatment* OR home-service* OR “home help” OR “domestic health care” OR “domestic healthcare” OR “public health nursing” OR “district nursing” OR “community nursing” OR “community based nursing” OR “Community Living” OR “Assisted Living” OR “Community Reintegration” OR “Home Occupational Therapy” OR “Home Physical Therapy” OR “Home Rehabilitation” OR “Respite Care” OR “Preventive Health Care” OR “Gerontologic Care”) )

## Concept 3: Falls prevention

((MH "Accidental Falls") OR TI(slip* OR fall*) OR AB(slip* OR fall*)) AND ((MH "Safety") OR TI(prevention* OR “preventive” OR "patient Safety") OR AB(prevention* OR “preventive” OR "patient Safety"))

## Total search strategy

( ( (MH "Aged") OR (MH "Aged, 80 and Over") OR (MH "Centenarians") OR (MH "Frail Elderly") OR (MH "Aging") OR (MH "Cognitive Aging") OR (MH "Longevity") ) OR ( TI(Aged* OR Advanced-age* OR Geriatric OR Senior* OR 65 OR 70 OR 75 OR 80 OR 85 OR 90 OR 95 OR 100 OR Advancing-year* OR Ageing OR Aging OR Geronto* OR Declining-year* OR Feeble* OR Of age OR Old-adult* OR Older-adult* OR Longevity OR “Ripe age” OR Senil* OR “65-75” OR “65-80” OR “65-85” OR “65-90” OR “65-95” OR “65-100” OR “70-75” OR “75-80” OR “75-85” OR “75-90” OR “75-95” OR “75-100” OR “80-85” OR “85-90” OR “85-95” OR “85-100” OR “90-95” OR “90-100” OR “95-100” OR Frail* OR Elder* OR Nonagerian* OR Octogenarian* OR Centarian* OR Senescence OR “Oldest-old”) OR AB(Aged* OR Advanced-age* OR Geriatric OR Senior* OR 65 OR 70 OR 75 OR 80 OR 85 OR 90 OR 95 OR 100 OR Advancing-year* OR Ageing OR Aging OR Geronto* OR Declining-year* OR Feeble* OR Of age OR Old-adult* OR Older-adult* OR Longevity OR “Ripe age” OR Senil* OR “65-75” OR “65-80” OR “65-85” OR “65-90” OR “65-95” OR “65-100” OR “70-75” OR “75-80” OR “75-85” OR “75-90” OR “75-95” OR “75-100” OR “80-85” OR “85-90” OR “85-95” OR “85-100” OR “90-95” OR “90-100” OR “95-100” OR Frail* OR Elder* OR Nonagerian* OR Octogenarian* OR Centarian* OR Senescence OR “Oldest-old”) ) ) AND ( ( (MH "Community Living") OR (MH "Assisted Living") OR (MH "Community Reintegration") OR (MH "Community Health Services") OR (MH "Community Health Nursing") OR (MH "Community Networks") OR (MH "Home Health Care") OR (MH "Home Nursing, Professional") OR (MH "Home Occupational Therapy") OR (MH "Home Physical Therapy") OR (MH "Home Rehabilitation") OR (MH "Respite Care") OR (MH "Preventive Health Care") OR (MH "Senior Centers") OR (MH "Housing for the Elderly") OR (MH "Ambulatory Care Facilities") OR (MH "Community Health Centers") OR (MH "Home Health Agencies") OR (MH "Home Nursing") OR (MH "Gerontologic Care") OR (MH "Homemaker Services") OR (MH "Community Medicine") OR (MH "Primary Health Care") OR (MH "Health Services for the Aged") OR (MH "Housing for the Elderly") ) OR ( TI(“Independent Living” OR Adult-Day-Care-Center* OR “Home Health Nursing” OR Community-Network* OR “Community Participation” OR “Home Nursing” OR Homemaker-Service* OR Senior-Cent* OR “Community Medicine” OR “Community-care” OR Ambulatory-Care-Facilit* OR “Primary Health Care” OR “Primary care” OR Home-health-servic* OR “Health Services for the Aged” OR “Housing for the Elderly” OR Community-Dwelling* OR “Aging in Place” OR Community-Health* OR “Home Health Care” OR “home-healthcare” OR Community-Involvement* OR “Public Participation” OR Community-Action* OR “Domiciliary Care” OR “Home Care” OR “Centers for the Aged” OR Ambulatory-Health-Cent* OR Neighborhood-Health-Cent* OR Neighbourhood-Health-Cent* OR “Primary Healthcare” OR Home-Health-Agenc* OR Geriatric-Health-Servic* OR Assisted-Living-Facilit* OR “Home visit” OR “visiting nursing service” OR outpatient-department* OR “elderly care” OR “old age assistance” OR “primary nursing care” OR “first line care” OR visiting-nursing-station* OR visiting-nurse-station* OR visiting-nurse-service* OR visiting-nursing-service* OR house-call* OR home-visit* OR home-health-agenc* OR “homecare” OR home-treatment* OR home-service* OR “home help” OR “domestic health care” OR “domestic healthcare” OR “public health nursing” OR “district nursing” OR “community nursing” OR “community based nursing” OR “Community Living” OR “Assisted Living” OR “Community Reintegration” OR “Home Occupational Therapy” OR “Home Physical Therapy” OR “Home Rehabilitation” OR “Respite Care” OR “Preventive Health Care” OR “Gerontologic Care”) OR AB(“Independent Living” OR Adult-Day-Care-Center* OR “Home Health Nursing” OR Community-Network* OR “Community Participation” OR “Home Nursing” OR Homemaker-Service* OR Senior-Cent* OR “Community Medicine” OR “Community-care” OR Ambulatory-Care-Facilit* OR “Primary Health Care” OR “Primary care” OR Home-health-servic* OR “Health Services for the Aged” OR “Housing for the Elderly” OR Community-Dwelling* OR “Aging in Place” OR Community-Health* OR “Home Health Care” OR “home-healthcare” OR Community-Involvement* OR “Public Participation” OR Community-Action* OR “Domiciliary Care” OR “Home Care” OR “Centers for the Aged” OR Ambulatory-Health-Cent* OR Neighborhood-Health-Cent* OR “Primary Healthcare” OR Home-Health-Agenc* OR Geriatric-Health-Servic* OR Assisted-Living-Facilit* OR “Home visit” OR “visiting nursing service” OR outpatient-department* OR “elderly care” OR “old age assistance” OR “primary nursing care” OR “first line care” OR visiting-nursing-station* OR visiting-nurse-station* OR visiting-nurse-service* OR visiting-nursing-service* OR house-call* OR home-visit* OR home-health-agenc* OR “homecare” OR home-treatment* OR home-service* OR “home help” OR “domestic health care” OR “domestic healthcare” OR “public health nursing” OR “district nursing” OR “community nursing” OR “community based nursing” OR “Community Living” OR “Assisted Living” OR “Community Reintegration” OR “Home Occupational Therapy” OR “Home Physical Therapy” OR “Home Rehabilitation” OR “Respite Care” OR “Preventive Health Care” OR “Gerontologic Care”) ) ) AND ( ((MH "Accidental Falls") OR TI(slip* OR fall*) OR AB(slip* OR fall*)) AND ((MH "Safety") OR TI(prevention* OR “preventive” OR "patient Safety") OR AB(prevention* OR “preventive” OR "patient Safety")) )

# WoS

## Concept 1: Older person

“aged*” OR “elder*” OR “senior*” OR “aging*” OR “senescence” OR “advanced age*” OR “geriatric” OR “65” OR “70” OR “75” OR “80” OR “85” OR “90” OR “95” OR “100” OR “advancing-year*” OR “ageing” OR “geronto*” OR “declining-year*” OR “feeble*” OR “of age” OR “old adult*” OR “older adult*” OR “ripe age” OR “senil*” OR “65-75” OR “65-80” OR “65-85” OR “65-90” OR “65-95” OR “65-100” OR “70-75” OR “75-80” OR “75-85” OR “75-90” OR “75-95” OR “75-100” OR “80-85” OR “85-90” OR “85-95” OR “85-100” OR “90-95” OR “90-100” OR “95-100” OR “frail*” OR “nonagerian*” OR “octogenarian*” OR “centarian*” OR “oldest-old” OR “longevity”

## Concept 2: Community setting

“independent living” OR “health service* for the elder*” OR “geriatric health service*” OR “neighborhood health cent*” OR “neighbourhood health cent*” OR “ambulatory health cent*” OR “community action*” OR “public participation” OR “community involvement*” OR “community dwelling*” OR “home health servic*” OR “primary care” OR “assisted living” OR “old age assistance” OR “elderly care” OR “health services for the aged” OR “primary nursing care” OR “primary healthcare” OR “first line care” OR “primary health care” OR “community health center*” OR “ambulatory care facilit*” OR “outpatient department*” OR “community medicine” OR “community participation” OR “community network*” OR “visiting nursing station*” OR “visiting nurse station*” OR “visiting nurse service*” OR “visiting nursing service*” OR “house call*” OR “home visit*” OR “home health agenc*” OR “homemaker service*” OR “homecare” OR “home treatment*” OR “home service*” OR “home nursing” OR “home help” OR “home health nursing” OR “home health care” OR “domiciliary care” OR “domestic health care” OR “domestic healthcare” OR “home care” OR “adult day care cent*” OR “senior cent*” OR “cent* for the elderly” OR “cent* for the aged” OR “public health nursing” OR “district nursing” OR “community nursing” OR “community based nursing” OR “community health*” OR “community care” OR “aging in place” OR “housing for the elderly” OR “home$healthcare” OR “community living” OR “assisted living” OR “community reintegration” OR “home rehabilitation” OR “respite care” OR “preventive health care” OR “gerontologic care”

## Concept 3: Falls prevention

(“fall*” OR “slip”) AND (“prevent*” OR “safet*”)

## Total search strategy

 (“aged*”  OR “elder*”  OR “senior*”  OR “aging*”  OR “senescence”  OR “advanced age*”  OR “geriatric”  OR “65”  OR “70”  OR “75”  OR “80”  OR “85”  OR “90”  OR “95”  OR “100”  OR “advancing-year*”  OR “ageing”  OR “geronto*”  OR “declining-year*”  OR “feeble*”  OR “of age”  OR “old adult*”  OR “older adult*”  OR “ripe age”  OR “senil*”  OR “65-75”  OR “65-80”  OR “65-85”  OR “65-90”  OR “65-95”  OR “65-100”  OR “70-75”  OR “75-80”  OR “75-85”  OR “75-90”  OR “75-95”  OR “75-100”  OR “80-85”  OR “85-90”  OR “85-95”  OR “85-100”  OR “90-95”  OR “90-100”  OR “95-100”  OR “frail*”  OR “nonagerian*”  OR “octogenarian*”  OR “centarian*”  OR “oldest-old”  OR “longevity”) AND (“independent living”  OR “health service* for the elder*”  OR “geriatric health service*”  OR “neighborhood health cent*”  OR “neighbourhood health cent*”  OR “ambulatory health cent*”  OR “community action*”  OR “public participation”  OR “community involvement*”  OR “community dwelling*”  OR “home health servic*”  OR “primary care”  OR “assisted living”  OR “old age assistance”  OR “elderly care”  OR “health services for the aged”  OR “primary nursing care”  OR “primary healthcare”  OR “first line care”  OR “primary health care”  OR “community health center*”  OR “ambulatory care facilit*”  OR “outpatient department*”  OR “community medicine”  OR “community participation”  OR “community network*”  OR “visiting nursing station*”  OR “visiting nurse station*”  OR “visiting nurse service*”  OR “visiting nursing service*”  OR “house call*”  OR “home visit*”  OR “home health agenc*”  OR “homemaker service*”  OR “homecare”  OR “home treatment*”  OR “home service*”  OR “home nursing”  OR “home help”  OR “home health nursing”  OR “home health care”  OR “domiciliary care”  OR “domestic health care”  OR “domestic healthcare”  OR “home care”  OR “adult day care cent*”  OR “senior cent*”  OR “cent* for the elderly”  OR “cent* for the aged”  OR “public health nursing”  OR “district nursing”  OR “community nursing”  OR “community based nursing”  OR “community health*”  OR “community care”  OR “aging in place”  OR “housing for the elderly”  OR “home$healthcare”  OR “community living”  OR “assisted living”  OR “community reintegration”  OR “home rehabilitation”  OR “respite care”  OR “preventive health care”  OR “gerontologic care”) AND ((“fall*”  OR “slip”)  AND (“prevent*”  OR “safet*”))

# Cochrane Library

## Concept 1: Older person

**Mesh-termen:**

**#1:** [mh "Aged"] OR [mh ^"Aging"] OR [mh "Cognitive Aging"] OR [mh "Longevity"]

**Vrije tekstwoorden:**

**#2:** (Aged* OR (Advanced NEXT age*) OR “Geriatric” OR Senior* OR 65 OR 70 OR 75 OR 80 OR 85 OR 90 OR 95 OR 100 OR (Advancing NEXT year*) OR “Ageing” OR “Aging” OR Geronto* OR (Declining NEXT year*) OR Feeble* OR “Of age” OR (Old NEXT adult*) OR (Older NEXT adult*) OR “Ripe age” OR Senil* OR 65?75 OR 65?80 OR 65?85 OR 65?90 OR 65?95 OR 65?100 OR 70?75 OR 75?80 OR 75?85 OR 75?90 OR 75?95 OR 75?100 OR 80?85 OR 85?90 OR 85?95 OR 85?100 OR 90?95 OR 90?100 OR 95?100 OR “Longevity” OR Frail* OR Elder* OR Nonagerian* OR Octogenarian* OR Centarian* OR “Senescence” OR “Oldest NEXT old”):ti,ab,kw

**#3:** #1 OR #2

## Concept 2: Community setting

**Mesh-termen:**

**#4:** [mh "Independent Living"] OR [mh ^"Community Health Services"] OR [mh "Adult Day Care Centers"] OR [mh ^"Community Health Nursing"] OR [mh "Home Health Nursing"] OR [mh "Community Networks"] OR [mh "Community Participation"] OR [mh ^"Home Care Services"] OR [mh "Home Nursing"] OR [mh "Homemaker Services"] OR [mh "Senior Centers"] OR [mh "Community Medicine"] OR [mh ^"Ambulatory Care Facilities"] OR [mh ^"Community Health Centers"] OR [mh ^"Primary Health Care"] OR [mh "Home Care Agencies"] OR [mh "Health Services for the Aged"] OR [mh "Assisted Living Facilities"] OR [mh "Housing for the Elderly"] OR [mh ^"Preventive Health Services"]

**Vrije tekstwoorden:**

**#5:** (“Independent Living” OR (Adult NEXT Day NEXT Care NEXT Center*) OR “Home Health Nursing” OR (Community NEXT Network*) OR “Community Participation” OR “Home Nursing” OR (Homemaker NEXT Service*) OR (Senior NEXT Cent*) OR “Community Medicine” OR (Community NEXT care) OR (Ambulatory NEXT Care NEXT Facilit*) OR “Primary Health Care” OR “Primary care” OR (Home NEXT health NEXT servic*) OR “Health Services for the Aged” OR “Housing for the Elderly” OR (Community NEXT Dwelling*) OR “Aging in Place” OR (Community NEXT Health*) OR “Home Health Care” OR (home NEXT healthcare) OR (Community NEXT Involvement*) OR “Public Participation” OR (Community NEXT Action*) OR “Domiciliary Care” OR “Home Care” OR “Centers for the Aged” OR (Ambulatory NEXT Health NEXT Cent*) OR (Neighborhood NEXT Health NEXT Cent*) OR (Neighbourhood NEXT Health NEXT Cent*) OR “Primary Healthcare” OR (Home NEXT Health NEXT Agenc*) OR (Geriatric NEXT Health NEXT Servic*) OR (Assisted NEXT Living NEXT Facilit*) OR “Home visit” OR “visiting nursing service” OR (outpatient NEXT department*) OR “elderly care” OR “old age assistance” OR “primary nursing care” OR “first line care” OR (visiting NEXT nursing NEXT station*) OR (visiting NEXT nurse NEXT station*) OR (visiting NEXT nurse NEXT service*) OR (visiting NEXT nursing NEXT service*) OR (house NEXT call*) OR (home NEXT visit*) OR (home NEXT health NEXT agenc*) OR homecare OR (home NEXT treatment*) OR (home NEXT service*) OR “home help” OR “domestic health care” OR “domestic healthcare” OR “public health nursing” OR “district nursing” OR “community nursing” OR “community based nursing” OR “Community living” OR “Assisted Living” OR “Community Reintegration” OR “Home Occupational Therapy” OR “Home Physical Therapy” OR “Home Rehabilitation” OR “Respite Care” OR (Preventive NEXT Health*) OR “Gerontologic Care”):ti,ab,kw

**#6:** #4 OR #5

## Concept 3: Falls prevention

#7: [mh "Accidental Falls"]

#8: (Fall* OR slip*):ti,ab,kw

#9: #7 OR #8

#10: [mh ^"Accident Prevention"] OR [mh ^"Safety"]

#11: (prevent* OR safet*):ti,ab,kw

#12: #10 OR #11

#13: #9 AND #12

## Total search strategy

**#3 AND #6 AND #13**
