# Supplementary material for: Strategies to implement multifactorial falls prevention interventions in community-dwelling older persons: a systematic review
Source: Implement Sci. 2023 Feb 6;18:4. doi: 10.1186/s13012-022-01257-w (PMC9901093; doi:10.1186/s13012-022-01257-w)
Supplement: Supplementary file 2 — Additional file 2. Sensitivity analysis. [file 13012_2022_1257_MOESM2_ESM.docx]

# Additional file 2: Sensitivity analysis

## Results of the Sensitivity analysis:

| **Study** | **Report** | **Relevance** | **Methodological quality** | **Sensitivity analysis** |
| --- | --- | --- | --- | --- |
| Study 1 | Clemson et al. 2004 | High | High | High |
|  | Ballinger et al. 2006 | High | High | High |
| Study 2 | Mackenzie et al. 2021 | High | High | High |
| Study 3 | Middlebrook et al. 2012 | High | Moderate | High |
| Study 4 | Mora Pinzon et al. 2019 | High | Moderate | High |
| Study 5 | Renehan et al. 2019 | High | Moderate | High |
| Study 6 | Garner et al. 1996 | High | Moderate | High |
|  | Hahn et al; 1996 | High | Moderate | High |
|  | Kempton et al. 2000 | High | Moderate | High |
|  | Barnett et al. 2003 | High | Moderate | High |
|  | Barnett et al. 2004 | High | Low | Moderate |
| Study 7 | Milisen et al. 2006 | High | Moderate | High |
| Study 8 | Mackenzie et al. 2020 | High | Moderate | High |
| Study 9 | Fortinsky et al. 2008 | High | Moderate | High |
| Study 10 | Gholamzadeh et al. 2021 | High | Moderate | High |
| Study 11 | Mahoney et al. 2016 | Moderate | Moderate | Moderate |
| Study 12 | Elley et al. 2008 | Moderate | Moderate | Moderate |
| Study 13 | Kramer et al. 2011 | Moderate | Moderate | Moderate |
| Study 14 | Zimmerman et al. 2017 | Moderate | Moderate | Moderate |
| Study 15 | Schlotthauer et al. 2017 | Moderate | Moderate | Moderate |
| Study 16 | Baker et al. 2007 | High | Low | Moderate |
| Study 17 | Kittipimpanon et al. 2012 | High | Low | Moderate |
| Study 18 | Tiedemann et al. 2021 | High | Low | Moderate |
| High + High = High  High + Moderate = High  Moderate + Moderate = Moderate High + Low = Moderate  Low + Low = Low | | | | |

## Relevance to the research question

| **Report** | **Is the implementation strategy clearly described?** | **Is the implementation strategy used in the community?** | **Is the evaluation of an implementation strategy for multifactorial falls prevention interventions in the community described?** | **Does this report measure the effectiveness of the implementation strategy?** | **Does this report explore the experiences with the implementation strategy for the implementation of multifactorial falls prevention interventions?** | **What is the relative contribution of this report to the SR (low, moderate, high)** |
| --- | --- | --- | --- | --- | --- | --- |
| Clemson et al. 2004 | + | ++ | + | + | ++ | High |
| Ballinger et al. 2006 | + | ++ | ++ | + | ++ | High |
| Mackenzie et al. 2021 | + | ++ | ++ | -- | ++ | High |
| Middlebrook et al. 2012 | + | ++ | ++ | -- | ++ | High |
| Mora Pinzon et al. 2019 | + | + | ++ | ++ | ++ | High |
| Renehan et al. 2019 | + | + | ++ | ++ | ++ | High |
| Garner et al. 1996 | + | ++ | ++ | ++ | -- | High |
| Hahn et al; 1996 | + | ++ | ++ | ++ | -- | High |
| Kempton et al. 2000 | + | ++ | + | ++ | -- | High |
| Barnett et al. 2003 | + | ++ | + | ++ | -- | High |
| Barnett et al. 2004 | + | ++ | + | ++ | -- | High |
| Milisen et al. 2006 | + | ++ | ++ | ++ | + | High |
| Mackenzie et al. 2020 | + | ++ | ++ | + | ++ | High |
| Fortinsky et al. 2008 | + | ++ | ++ | + | -- | High |
| Gholamzadeh et al. 2021 | + | ++ | ++ | + | -- | High |
| Mahoney et al. 2016 | + | + | ++ | -- | -- | Moderate |
| Elley et al. 2008 | + | ++ | + | + | -- | Moderate |
| Kramer et al. 2011 | - | + | + | + | ++ | Moderate |
| Zimmerman et al. 2017 | + | + | - | -- | + | Moderate |
| Schlotthauer et al. 2017 | + | ++ | ++ | + | - | Moderate |
| Baker et al. 2007 | + | ++ | ++ | -- | ++ | High |
| Kittipimpanon et al. 2012 | + | ++ | ++ | + | + | High |
| Tiedemann et al. 2021 | + | ++ | ++ | ++ | + | High |
| -- = Not described in the report. - = Described in the report. However, it is not clearly described.  + = Described in the report. However it is not the main purpose of the report and not in detail described. ++ = Described in the report. It is the main purpose of the report.  Low = If at least three items were scored with '- -' OR '- -' on question 'Is the implementation strategy described'. Moderate = If mainly + or - was used.  High = If mainly ++ and + was used | | | | | | |

## Mixed Methods Appraisal Tool (different designs)

| **Report** | **Design** | **Is there an adequate rationale for using a mixed methods design to address the research question?** | **Are the different components of the study effectively integrated to answer the research question?** | **Are the outputs of the integration of qualitative and quantitative components adequately interpreted?** | **Are divergences and inconsistencies between quantitative and qualitative results adequately addressed?** | **Do the different components of the study adhere to the quality criteria of each tradition of the methods involved?** | **Quality of the study** |
| --- | --- | --- | --- | --- | --- | --- | --- |
| Barnett et al. 2004 | Mixed Methods Study | Yes | Can't Tell | Can't Tell | No | Can't Tell | Low Quality |
| Kittipimpanon et al. 2012 | Mixed Methods Study | Yes | Can't Tell | Can't Tell | No | No | Low Quality |
| Mackenzie et al. 2020 | Mixed Methods Study | Yes | Can't Tell | Can't Tell | Yes | No | Moderate Quality |
| Milisen et al. 2006 | Mixed Methods Study | Yes | Yes | Can't Tell | No | No | Moderate Quality |
| Mora Pinzon et al. 2019 | Mixed Methods Study | Yes | Can't Tell | Yes | Yes | No | Moderate Quality |
| Renehan et al. 2019 | Mixed Methods Study | Yes | Yes | No | Yes | No | Moderate Quality |
| Schlotthauer et al. 2017 | Mixed Methods Study | Yes | Can't Tell | Can't Tell | Yes | No | Moderate Quality |
| Zimmerman et al. 2017 | Mixed Methods Study | Yes | Yes | Can't Tell | Yes | No | Moderate Quality |
| Low: ≥ 0 times 'yes' Moderate: ≥ 2 times 'yes' High: ≥ 4 times 'yes' | | | | | | | |

| **Report** | **Design** | **Is the qualitative approach appropriate to answer the research question?** | **Are the qualitative data collection methods adequate to address the research question?** | **Are the findings adequately derived from the data?** | **Is the interpretation of results sufficiently substantiated by data?** | **Is there coherence between qualitative data sources, collection, analysis and interpretation?** | **Quality of the study** |
| --- | --- | --- | --- | --- | --- | --- | --- |
| Baker et al. 2007 | Qualitative Study | Yes | Can't Tell | Can't Tell | Can't Tell | Can't Tell | Low Quality |
| Ballinger et al. 2006 | Qualitative Study | Yes | Yes | Yes | Yes | Yes | High Quality |
| Kramer et al. 2011 | Qualitative Study | Yes | Yes | Can't Tell | Can't Tell | Yes | Moderate Quality |
| Mahoney et al. 2016 | Qualitative Study | Yes | Yes | Can't Tell | Can't Tell | Yes | Moderate Quality |
| Middlebrook et al. 2012 | Qualitative Study | Yes | Yes | Can't Tell | Can't Tell | Yes | Moderate Quality |
| Mackenzie et al. 2021 | Qualitative study | Yes | Yes | Can't Tell | Yes | Yes | High Quality |
| Low: ≥ 0 times 'yes' Moderate: ≥ 2 times 'yes' High: ≥ 4 times 'yes' | | | | | | | |

| **Report** | **Design** | **Is the sampling strategy relevant to address the research question?** | **Is the sample representative of the target population?** | **Are the measurements appropriate?** | **Is the risk of nonresponse bias low?** | **Is the statistical analysis appropriate to answer the research question?** | **Quality of the study** |
| --- | --- | --- | --- | --- | --- | --- | --- |
| Barnett et al. 2003 | Quantitative Descriptive Study | Yes | No | Yes | No | Yes | Moderate Quality |
| Fortinsky et al. 2008 | Quantitative Descriptive Study | Yes | No | No | No | Yes | Moderate Quality |
| Garner et al. 1996 | Quantitative Descriptive Study | Yes | Can't Tell | Yes | Can't Tell | Yes | Moderate Quality |
| Low: ≥ 0 times 'yes' Moderate: ≥ 2 times 'yes' High: ≥ 4 times 'yes' | | | | | | | |

| **Report** | **Design** | **Are the participants representative of the target population?** | **Are measurements appropriate regarding both the outcome and intervention (or exposure)?** | **Are there complete outcome data?** | **Are the confounders accounted for in the design and analysis?** | **During the study period, is the intervention administered (or exposure occurred) as intended?** | **Quality of the study** |
| --- | --- | --- | --- | --- | --- | --- | --- |
| Hahn et al. 1996 | Quantitative non-RCT Study | Yes | Yes | No | Yes | Can't Tell | Moderate Quality |
| Kempton et al. 2000 | Quantitative non-RCT Study | Can't Tell | Yes | Yes | Yes | Can't Tell | Moderate Quality |
| Tiedemann et al. 2021 | Quantitative non-RCT Study | Yes | No | No | No | No | Low Quality |
| Low: ≥ 0 times 'yes' Moderate: ≥ 2 times 'yes' High: ≥ 4 times 'yes' | | | | | | | |

| **Report** | **Design** | **Is randomization appropriately performed?** | **Are the groups comparable at baseline?** | **Are there complete outcome data?** | **Are outcome assessors blinded to the intervention provided?** | **Did the participants adhere to the assigned intervention?** | **Calculated Quality** |
| --- | --- | --- | --- | --- | --- | --- | --- |
| Clemson et al. 2004 | Quantitative RCT Study | Yes | Yes | Yes | Yes | No | High Quality |
| Elley et al. 2008 | Quantitative RCT Study | Yes | Yes | Can't Tell | Yes | No | Moderate Quality |
| Gholamzadeh et al. 2021 | Quantitative RCT Study | Yes | Yes | Yes | No | Can’t tell | Moderate Quality |
| Low: ≥ 0 times 'yes' Moderate: ≥ 2 times 'yes' High: ≥ 4 times 'yes' | | | | | | | |
