# Supplementary material for: Strategies to implement multifactorial falls prevention interventions in community-dwelling older persons: a systematic review
Source: Implement Sci. 2023 Feb 6;18:4. doi: 10.1186/s13012-022-01257-w (PMC9901093; doi:10.1186/s13012-022-01257-w)
Supplement: Supplementary file 3 — Additional file 3. Table Implementation strategies. [file 13012_2022_1257_MOESM3_ESM.docx]

Table: Behavioral change methods (individual level).

|  |  |  | **Basic Methods** | | | | | | | | | | | **Know-ledge** | | **Aware-ness and risk perception** | | **Habitual, Automatic and Impulsive behaviors** | | | **Attitudes, beliefs, outcome expectations** | | | | **Social influence** | | **Skills, capabilities and self-efficacy** | | | **Public Stigma** |
| --- | --- | --- | --- | --- | --- | --- | --- | --- | --- | --- | --- | --- | --- | --- | --- | --- | --- | --- | --- | --- | --- | --- | --- | --- | --- | --- | --- | --- | --- | --- |
|  | **Program** | **Target population** | **Participation** | **Belief selection** | **Persuasive communication** | **Active learning** | **Tailoring** | **Individualization** | **Modeling** | **Feedback** | **Reinforcement** | **Motivational interviewing** | **Facilitation** | **Discussion** | **Elaboration** | **Consciousness raising** | **Personalize risk** | **Implementation intentions** | **Planning coping responses** | **Public commitment** | **Direct experience** | **Elaboration** | **Repeated exposure** | **Cultural similarity** | **Mobilizing social support** | **Provide opportunities for social comparison** | **Guided practice** | **Goal setting** | **Planning coping responses** | **Cooperative learning** |
| **Study 1** Clemson, 2004 Ballinger, 2006 | Stepping On | OP | X |  |  | X | X | X |  | X | X |  | X | X | X | X | X | X | X |  | X | X |  |  | X | X | X | X | X | X |
|  |  | HC |  |  |  | X | X |  |  | X |  |  |  |  |  |  |  |  |  |  |  |  |  |  |  |  | X |  |  |  |
| **Study 2** Mackenzie et al. 2021 | iSOLVE | OP |  |  |  |  | X |  |  |  |  |  |  |  |  | X |  |  |  |  |  |  |  |  |  |  |  |  |  |  |
|  |  | HC |  |  |  | X | X |  |  |  |  |  |  |  |  | X |  |  |  |  |  |  |  |  |  |  | X |  |  |  |
| **Study 3** Middlebrook, 2012 | Chronic Disease Management | OP |  |  |  |  | X | X |  |  |  |  |  |  |  |  | X |  |  |  |  |  |  |  |  |  |  |  |  |  |
|  |  |  |  |  |  |  |  |  |  |  |  |  |  |  |  |  |  |  |  |  |  |  |  |  |  |  |  |  |  |  |
| **Study 4** Mora-Pinzon, 2019 | Stepping On | OP | X |  |  | X | X | X |  | X | X |  | X | X | X | X | X | X | X |  | X | X |  | X | X | X | X | X | X | X |
|  |  | HC |  |  |  | X | X |  |  | X |  |  |  |  |  |  |  |  |  |  |  |  |  | X |  |  | X |  |  |  |
|  |  |  |  |  |  |  |  |  |  |  |  |  |  |  |  |  |  |  |  |  |  |  |  |  |  |  |  |  |  |  |
| **Study 5** Renehan, 2019 | Posthospital fall prevention intervention | OP |  |  | X | X | X | X |  |  |  |  |  |  | X |  | X |  | X |  |  | X |  |  |  |  |  | X | X |  |
| **Study 6** Garner, 1996 Hahn, 1996 Kempton, 2000 Barnett, 2003 Barnett, 2004 | Stay On your Feet | OP | X | X | X | X | X |  | X |  |  |  |  |  | X | X | X |  |  |  |  | X | X |  | X |  |  |  |  |  |
|  |  | HC |  | X | X |  | X |  |  |  |  |  |  |  |  | X |  |  | X |  |  |  | X |  |  |  |  |  | X |  |
|  |  | OR |  | X | X |  | X |  |  |  |  |  |  |  |  | X |  |  |  |  |  |  | X |  |  |  |  |  |  |  |
|  |  | CO |  | X | X |  | X |  |  |  |  |  |  |  |  | X |  |  |  |  |  |  | X |  |  |  |  |  |  |  |
|  |  | PO/SO |  | X | X |  | X |  |  |  |  |  |  |  |  | X |  |  |  |  |  |  | X |  |  |  |  |  |  |  |
| **Study 7** Milisen, 2006 | Nurse-led multifactorial fall prevention intervention | OP | X |  |  | X | X | X |  |  |  |  |  |  |  | X | X |  |  |  |  |  |  |  |  |  |  |  |  |  |
| **Study 8** Mackenzie, 2020 | Chronic Disease Management | OP |  |  |  |  | X | X |  | X |  |  |  |  |  |  | X | X | X |  |  |  |  |  |  |  |  |  | X |  |
|  |  | HC |  |  |  | X |  |  |  |  |  |  |  |  |  |  |  |  |  |  |  |  |  |  |  |  |  |  |  |  |
| **Study 9** Fortinsky, 2008 | Support for implementation & education of HC | OP |  |  |  |  | X |  |  |  |  | X |  |  |  |  | X |  |  |  |  |  |  |  |  |  |  |  |  |  |
|  |  | HC |  |  |  | X |  |  | X |  |  |  |  |  |  |  |  |  |  |  |  |  |  |  |  |  |  |  |  |  |
|  |  |  |  |  |  |  |  |  |  |  |  |  |  |  |  |  |  |  |  |  |  |  |  |  |  |  |  |  |  |  |
| **Study 10** Gholamzadeh et al. 2021 | Stepping on | OP | X |  |  | X | X | X |  | X | X |  | X | X | X | X | X | X | X |  | X | X |  |  | X | X | X | X | X | X |
| **Study 11** Mahoney, 2016 | Stepping On | OP | X |  |  | X | X | X |  | X | X |  | X | X | X | X | X | X | X |  | X | X |  |  | X | X | X | X | X | X |
|  |  | HC |  |  |  | X | X |  |  | X |  |  |  |  |  |  |  |  |  |  |  |  |  |  |  |  | X |  |  |  |
| **Study 12** Elley, 2008 | Nurse-led multifactorial fall prevention intervention | OP |  |  |  |  | X | X |  |  |  |  |  |  |  |  | X |  |  |  |  |  |  |  |  |  |  |  |  |  |
|  |  | HC |  |  |  | X |  |  |  |  |  |  |  |  |  |  |  |  |  |  |  |  |  |  |  |  |  |  |  |  |
| **Study 13** Kramer, 2011 | InSTEP | OP |  |  |  | X | X |  |  |  |  |  |  |  |  |  | X |  |  |  |  |  |  |  | X |  |  |  |  |  |
|  |  | HC |  |  |  | X |  |  |  |  |  |  |  |  |  |  |  |  |  |  |  |  |  |  |  |  |  |  |  |  |
|  |  |  |  |  |  |  |  |  |  |  |  |  |  |  |  |  |  |  |  |  |  |  |  |  |  |  |  |  |  |  |
| **Study 14** Zimmerman, 2017 | Assisted Living Falls Prevention and Monitoring Program (AL-FPMP) | HC |  |  |  | X | X |  | X | X |  |  |  |  |  |  |  |  |  | X |  |  |  |  |  |  |  |  |  |  |
|  |  | OR |  |  |  |  | X |  |  | X |  |  |  |  |  |  |  |  |  |  |  |  |  |  |  |  |  |  |  |  |
| **Study 15** Schlotthauer, 2017 | Stepping On | OP | X |  |  | X | X | X |  | X | X |  | X | X | X | X | X | X | X |  | X | X |  |  | X | X | X | X | X | X |
|  |  | HC |  |  |  | X | X |  |  | X |  |  |  |  |  |  |  |  |  |  |  |  |  |  |  |  | X |  |  |  |
| **Study 16** Baker, 2007 | Step by step | OP | X | X | X |  | X | X | X |  |  | X | X |  |  | X | X |  |  |  |  |  |  | X |  |  |  |  |  |  |
|  |  | HC | X |  |  | X | X |  | X | X |  |  |  |  |  |  |  |  |  |  |  |  |  |  |  |  | X |  |  |  |
|  |  | OR |  |  |  |  | X |  |  |  |  |  |  |  |  |  |  |  |  |  |  |  |  |  |  |  |  |  |  |  |
| **Study 17** Kittipimpanon, 2012 | Community based fall-prevention program | OP | X | X |  | X | X |  |  |  |  |  |  | X |  | X | X |  |  |  |  |  |  |  | X |  |  |  |  |  |
|  |  | OR |  |  |  |  |  |  |  |  |  |  |  |  |  | X |  |  |  |  |  |  |  |  |  |  |  |  |  |  |
|  |  | CO |  | X |  | X | X |  |  |  |  |  |  |  |  | X |  |  |  |  |  |  |  |  |  |  |  |  |  |  |
| **Study 18**  Tiedemann, 2021 | Stepping On | OP | X |  |  | X | X | X |  | X | X |  | X | X | X | X | X | X | X |  | X | X |  |  | X | X | X | X | X | X |
|  |  | HC |  |  |  | X | X |  |  | X |  |  |  |  |  |  |  |  |  |  |  |  |  |  |  |  | X |  |  |  |
| OP = Older Person  HC = Health care professional  OR = Organization  CO = Community  PO/SO = Policy/society | | | | | | | | | | | | | | | | | | | | | | | | | | | | | | |

Table: Behavioral change methods (environmental level).

|  |  | **Basic methods** | | | **Social Norms** | | **Social support and social networks** | | **Change organizations** | **Change communities** | | **Policy** | |
| --- | --- | --- | --- | --- | --- | --- | --- | --- | --- | --- | --- | --- | --- |
|  | **Program** | **Participatory problem solving** | **Modeling** | **Technical assistance** | **Mass-media role-modeling** | **Mobilizing social networks** | **Enhancing network linkages** | **Use of lay health workers, peer education** | **Increasing stakeholder influence** | **community assessment** | **Community development** | **Forming coalitions** | **Agenda setting** |
| **Study 1** Clemson, 2004  Ballinger, 2006 | Stepping On |  |  | X |  |  |  | X |  |  |  |  |  |
|  |  |  |  |  |  |  |  |  |  |  |  |  |  |
| **Study 2** Mackenzie et al. 2021 | iSOLVE |  |  | X |  |  | X |  | X |  |  | X |  |
| **Study 3** Middlebrook, 2012 | Chronic Disease Management |  |  | X |  |  |  |  | X |  |  | X |  |
|  |  |  |  |  |  |  |  |  |  |  |  |  |  |
| **Study 4** Mora-Pinzon, 2019 | Stepping On | X |  | X |  |  |  | X |  |  |  |  |  |
|  |  |  |  |  |  |  |  |  |  |  |  |  |  |
|  |  |  |  |  |  |  |  |  |  |  |  |  |  |
| **Study 5** Renehan, 2019 | Posthospital fall prevention intervention |  |  |  |  |  |  |  |  |  |  |  |  |
| **Study 6** Garner, 1996  Hahn, 1996  Kempton, 2000  Barnett, 2003  Barnett, 2004 | Stay On your Feet | X |  | X | X | X | X | X | X |  | X | X | X |
|  |  |  |  |  |  |  |  |  |  |  |  |  |  |
|  |  |  |  |  |  |  |  |  |  |  |  |  |  |
|  |  |  |  |  |  |  |  |  |  |  |  |  |  |
|  |  |  |  |  |  |  |  |  |  |  |  |  |  |
| **Study 7** Milisen, 2006 | Nurse-led multifactorial fall prevention intervention |  |  |  |  |  |  |  |  |  |  |  |  |
| **Study 8** Mackenzie, 2020 | Chronic Disease Management |  |  | X |  |  |  |  | X |  |  | X |  |
|  |  |  |  |  |  |  |  |  |  |  |  |  |  |
| **Study 9** Fortinsky, 2008 | Support for implementation & education of healthcare providers |  | X | X |  |  |  | X |  |  |  |  |  |
|  |  |  |  |  |  |  |  |  |  |  |  |  |  |
|  |  |  |  |  |  |  |  |  |  |  |  |  |  |
| **Study 10** Gholamzadeh et al. 2021 | Stepping On |  |  |  |  |  |  |  |  |  |  |  |  |
| **Study 11** Mahoney, 2016 | Stepping On | X |  | X |  |  |  | X |  |  |  |  |  |
|  |  |  |  |  |  |  |  |  |  |  |  |  |  |
| **Study 12** Elley, 2008 | Nurse-led multifactorial fall prevention intervention |  |  | X |  |  |  |  |  |  |  |  |  |
|  |  |  |  |  |  |  |  |  |  |  |  |  |  |
| **Study 13** Kramer, 2011 | InSTEP | X |  | X |  |  |  |  | X |  |  | X |  |
|  |  |  |  |  |  |  |  |  |  |  |  |  |  |
|  |  |  |  |  |  |  |  |  |  |  |  |  |  |
| **Study 14** Zimmerman, 2017 | Assisted Living Falls Prevention and Monitoring Program (AL-FPMP) | X | X | X |  |  | X | X | X |  |  | X |  |
|  |  |  |  |  |  |  |  |  |  |  |  |  |  |
| **Study 15** Schlotthauer, 2017 | Stepping On |  |  | X |  |  |  | X |  |  |  |  |  |
| **Study 16** Baker, 2007 | Step by step | X | X | X |  |  |  | X | X | X | X | X |  |
|  |  |  |  |  |  |  |  |  |  |  |  |  |  |
| **Study 17** Kittipimpanon, 2012 | Community based fall-prevention program | X |  |  |  | X | X | X | X | X | X | X |  |
|  |  |  |  |  |  |  |  |  |  |  |  |  |  |
|  |  |  |  |  |  |  |  |  |  |  |  |  |  |
| **Study 18**  Tiedemann, 2021 | Stepping On |  |  | X |  |  |  | X |  |  |  |  |  |
|  |  |  |  |  |  |  |  |  |  |  |  |  |  |
|  |  |  |  |  |  |  |  |  |  |  |  |  |  |
